# Keratinocytes Determine Th1 Immunity during Early Experimental Leishmaniasis

**DOI:** 10.1371/journal.ppat.1000871

**Published:** 2010-04-29

**Authors:** Jan M. Ehrchen, Kirsten Roebrock, Dirk Foell, Nadine Nippe, Esther von Stebut, Johannes M. Weiss, Niels-Arne Münck, Dorothee Viemann, Georg Varga, Carsten Müller-Tidow, Hans-Joachim Schuberth, Johannes Roth, Cord Sunderkötter

**Affiliations:** 1 Institute of Immunology, University of Muenster, Muenster, Germany; 2 Department of Dermatology, University of Muenster, Muenster, Germany; 3 IZKF Münster, University of Muenster, Muenster, Germany; 4 Department of Dermatology, University of Mainz, Mainz, Germany; 5 Department of Dermatology, University of Ulm, Ulm, Germany; 6 Department of Pediatrics, University of Muenster, Muenster, Germany; 7 Department of Medicine, Hematology and Oncology, University of Muenster, Muenster, Germany; 8 Institute of Immunology, University of Veterinary Medicine, Hannover, Germany; Imperial College London, United Kingdom

## Abstract

Experimental *leishmaniasis* is an excellent model system for analyzing Th1/Th2 differentiation. Resistance to *Leishmania (L.) major* depends on the development of a *L. major* specific Th1 response, while Th2 differentiation results in susceptibility. There is growing evidence that the microenvironment of the early affected tissue delivers the initial triggers for Th-cell differentiation. To analyze this we studied differential gene expression in infected skin of resistant and susceptible mice 16h after parasite inoculation. Employing microarray technology, bioinformatics, laser-microdissection and *in-situ*-hybridization we found that the epidermis was the major source of immunomodulatory mediators. This epidermal gene induction was significantly stronger in resistant mice especially for several genes known to promote Th1 differentiation (IL-12, IL-1β, osteopontin, IL-4) and for IL-6. Expression of these cytokines was temporally restricted to the crucial time of Th1/2 differentiation. Moreover, we revealed a stronger epidermal up-regulation of IL-6 in the epidermis of resistant mice. Accordingly, early local neutralization of IL-4 in resistant mice resulted in a Th2 switch and mice with a selective IL-6 deficiency in non-hematopoietic cells showed a Th2 switch and dramatic deterioration of disease. Thus, our data indicate for the first time that epidermal cytokine expression is a decisive factor in the generation of protective Th1 immunity and contributes to the outcome of infection with this important human pathogen.

## Author Summary

To clear skin infections with the parasite *Leishmania major*, a specific T-helper (Th)-cell immune response has to be generated. The type of Th-cell response is determined early after infection by yet unknown mechanisms. In resistant mice a Th1-pattern is generated. A Th2-pattern in BALB/c mice, however, results in susceptibility. An analysis of these mechanisms is important for a better understanding of both host-parasite interactions and non-infectious Th-cell driven inflammatory skin disorders (e.g. atopic dermatitis). We analyzed how the infected skin influenced the Th-cell response. Therefore, we compared gene-expression early after infection in the skin of resistant and susceptible mice. Several cytokines (like IL-1β, IL-12, osteopontin, IL-4 and IL-6) were more strongly produced in the skin of resistant mice and therefore could be important for Th1-differentiation. We demonstrated that they were expressed by epidermal keratinocytes. Using mice with a deficiency for IL-6 in keratinocytes but not in immune cells and by inhibiting the action of early produced IL-4 we revealed that keratinocyte-derived IL-6 and IL-4 are important for resistance against *Leishmania*. Thus, our results indicate that the epidermis controls Th1-differentiation and may be a new pharmacological target for modification of Th-differentiation.

## Introduction

Experimental leishmaniasis has been the first model to directly demonstrate the relevance of the T helper 1/T helper 2 (Th1/Th2) dichotomy for the outcome of an infection or disease *in vivo*. Upon cutaneous infection with *L. major*, C57BL/6 mice generate a Th1 response and subsequently control infection, mainly due to activation of macrophages by IFN-γ. BALB/c mice develop a Th2 response and succumb to progressive disease (for a review see [1]).

The decisive events for the development of a Th1 or Th2 response take place early after infection - most likely during the first two days - since an influence on Th1/2 differentiation can only be achieved by pharmacological manipulation in this critical time frame [1]. The regulatory mechanisms responsible for this differentiation have long been supposed to primarily occur in the lymph node [1]. However, the molecular and cellular mechanisms preceding the immunological mechanism in the lymph nodes are not identified so far.

During the last years it became increasingly clear that the skin as the site of primary infection also influences the direction of the immune response. We and others have demonstrated that already 2 days after infection there is a higher percentage of granulocytes in the infiltrate of BALB/c compared to C57BL/6 mice and that antibody-mediated elimination of these cells in susceptible mice results in a Th1 response and restoration of resistance [2], [3].

These differences in granulocyte infiltration point to important differences in the microenvironment of the infected tissue within the first hours of infection, which can be decisive for the direction of the T cell response. Accordingly, a change in the route or site of infection, e.g. via blood stream or nasal mucosa instead of skin, results in a Th2 response and non-healing disease in originally resistant mice [4], [5] confirming a relevant influence of the local environment of the skin on T-cell priming in experimental leishmaniasis.

The infected tissue has been suggested to generate signals within the first hours after inoculation of parasites which are then integrated and transferred via dendritic cells (DCs) to T-cells in draining lymph nodes where they induce Th1 or Th2 differentiation. These signals have been termed “tissue” or “danger” signals [6]–[8]. The types of such signals at the site of infection are as manifold as their potential cellular sources since they could derive from either resident or infiltrating cells.

Therefore our aim was to perform a global search for such early tissue signals at the site of infection which have the potential to influence the specific immune response.

Using microarray technology and bioinformatics we confirmed the existence of tissue signals which appear within the first hours at the site of infection and identified for the first time the epidermis as an important source for these signals. Among these signals we identified chemokines for macrophage recruitment as well as several cytokines with the potential to induce Th1 response (e.g. via influencing DC) which showed a stronger expression in the epidermis of resistant mice. Such cytokines included IL-4 and IL-6. While early neutralization of IL-4 resulted in a Th2 switch in originally resistant mice, also early production of IL-6 in the skin was revealed as a novel decisive factor in the generation of protective Th1 immunity. Thus, we present strong evidence that early activation of epidermal cells influences the resulting T-cell response against *L. major*.

## Results

### Screening by gene array and PCR for genes regulated in the early phase of experimental leishmaniasis

To test our hypothesis that immunomodulatory mediators relevant for defense against infection with *L. major* and the generation of a Th1/Th2 response are expressed early at the site of initial infection, we screened for gene expression patterns using both global microarray analysis as well as the more sensitive real time PCR technique.

Employing microarray technology we detected significant up-regulation of 189 genes and down-regulation of 16 genes in both mouse strains 16 hours after infection (Table S1, S2). The total number of regulated genes was greater in resistant than in susceptible mice (205 vs. 146 genes). While only 4 genes were regulated significantly stronger in susceptible BALB/c mice, 59 genes were regulated significantly stronger in resistant C57BL/6 mice (Table 1 and Table S3, S4) and encompassed genes with a well known function in Th1/Th2 differentiation (such as IL-1β and osteopontin (opn)) [9], [10].

**Table 1:** Genes with differential regulation in C57BL/6 or BALB/c mice upon *L. major* infection.

Genesymbol	Description (NCBI Gene)	N-fold BALB/c	N-fold C57BL/6	p-value
**Chemokines and receptors**				
*Cxcl2*	chemokine (C-X-C motif) ligand 2	12.2	23.9	0.003
*Ccl9*	chemokine (C-C motif) ligand 9	1.9	3.7	0.008
*Spp1*	secreted phosphoprotein 1 (osteopontin)	4.8	28.3	0.001
*Ccr5*	chemokine (C-C motif) receptor 5	8.1	142.5	0.019
**Cytokines and related molecules**				
*IL-1b*	interleukin 1 beta	9.8	39.2	0.033
*Tgfb1*	transforming growth factor, beta 1	2.0	9.3	0.048
*S100a8*	S100 calcium binding protein A8	5.7	18.8	<0.001
*S100a9*	S100 calcium binding protein A9	8.3	24.6	0.001
*Ptx3*	pentaxin related gene	5.8	3.2	0.028
*Ifi202b*	interferon activated gene 202B	5.2	−1.8	0.001
**Other Genes involved in immune response**				
*Slpi*	Secretory leukocyte protease inhibitor	2.7	10.4	0.002
*Chi3l3*	chitinase 3-like 3 (Ym-1)	3.0	14.8	<0.001
*Saa3*	serum amyloid A 3	7.2	28.1	0.037
*Tnfrsf1b*	TNF receptor superfamily, member 1b	3.7	7.8	<0.001
*CD14*	CD14 antigen	2.2	5.3	0.003
*Temt*	thioether S-methyltransferase	−3.4	−6.5	0.002
**Genes involved in keratinocyte differentiation**				
*Sprr2B*	small proline-rich protein 2B	1.6	5.2	<0.001
*Sprr2a*	small proline-rich protein 2A	2.3	6.3	0.007
*Sprr2h*	small proline-rich protein 2H	−1.3	−2.9	0.005

**Figure 1:**
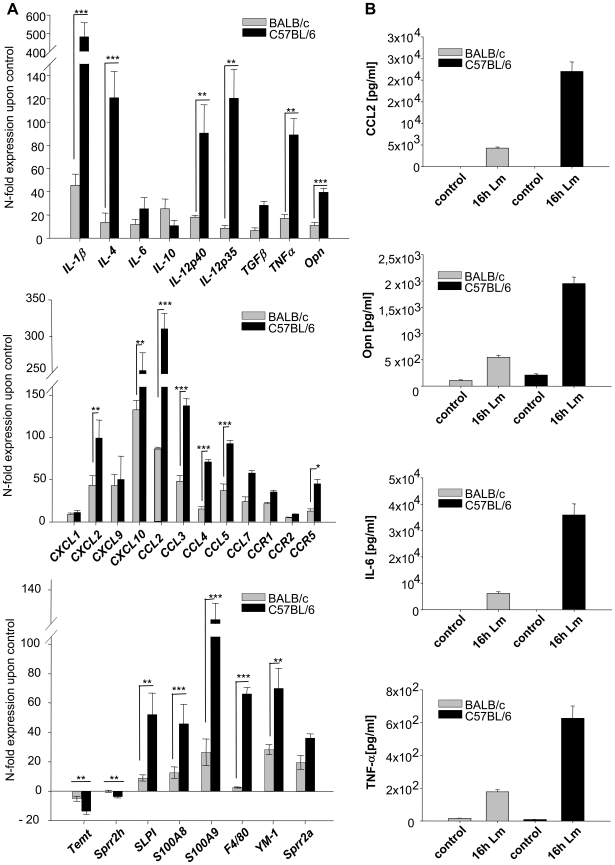
Analysis of gene and protein expression in *L. major* infected skin. **A**) RT-PCR Analysis of gene expression in 16 h *L. major* infected skin. The PCR data were normalized to GAPDH expression and mean N-fold regulation and SEM in comparison to PBS injected controls was calculated (n = 3). Grey bars: Regulation in BALB/c mice; Black bars: Regulation in C57BL/6 mice. * = *P*<0.05, ** = *P*<0.01, *** = *P*<0,001 for differences between C57BL/6 and BALB/c mice, Student's t-test. **B**) Protein secretion in *L. major* infected skin. Soluble CCL2, Opn, TNFα (16h after infection) and IL-6 (8h after infection) protein was measured in infected foots as described in Materials and Methods. Striped grey bars: Mean and SE in BALB/c mice; Black bars: Mean and SE C57BL/6 mice. The experiment was performed three times with similar results.

Considering the fact that whole tissue samples were analyzed one has to take into account that some genes could be highly regulated in a fraction of cells, while their absolute expression levels could still remain under the detection limit of the microarray analysis. Thus, the much more sensitive real-time PCR was applied i) to confirm the microarray data for selected genes ii) to extend the gene expression analysis especially to immunomodulatory mediators with known or suspected influence on Th1/2 differentiation whose expression was not detected by the less sensitive microarray analysis (Figure 1A and Table S5). PCR not only confirmed the induction of genes revealed by microarray analysis, but due to the higher sensitivity of the PCR analysis an up regulation of several chemokines and of the cytokines IL-12, TNFα, IL-4, and IL-6 could also be detected. All these genes were more strongly induced in C57BL/6 than in BALB/c.

For selected genes, among them those with suspected influence on Th1/2 differentiation (such as opn, TNFα, IL-6), we confirmed both expression on protein level and higher up-regulation in C57BL/6 mice by immunoprecipitation as well as cytometric bead assay (Figure 1B).

### Functional clustering

Besides cytokines many other genes were regulated in the skin in response to *L. major*. To get some functional insight into the pattern of regulated genes we applied an automated unbiased functional clustering using GENMAPP software [11], [12] and gene ontology annotations to determined which functional clusters among the regulated genes were statistically overrepresented. Accordingly, we found a statistically significant overrepresentation of genes involved in “inflammatory response”, “chemotaxis” and “cytokines” in the group of genes up-regulated in both mouse strains (Table S6, S7).

In agreement with the PCR-data we detected a significant over-representation of genes involved in “chemotaxis” and in “cell-mediated immune response” among those genes which were significantly stronger regulated in resistant mice. Importantly, genes involved in keratinocyte differentiation were also overrepresented in this group of genes (Table 2). These data indicate that gene expression of keratinocytes is markedly influenced early after infection with *L. major*.

**Table 2:** Functional gene clusters overrepresented.

Functional gene clusters overrepresented among genes stronger regulated in C57BL/6 mice	Z- Score	P-value	% of selection	% of all
**molecular function**				
cytokine activity	3.4	0.008	10.0	1.9
signal transducer activity	2.9	0.006	40.0	18.2
**biological process**				
immune response	11.4	<0.001	57.5	7.1
chemotaxis	11.3	<0.001	22.5	1.2
neutrophil chemotaxis	11.5	<0.001	7.5	0.1
humoral immune response	8.8	<0.001	20.0	1.5
keratinization	8.2	0.001	7.5	0.3
phagocytosis	8.2	<0.001	7.5	0.3
lymphocyte activation	6.3	0.001	15.0	1.6
cellular defense response	3.6	0.016	7.5	1.1
cell adhesion	3.0	0.004	15.0	4.3
signal transduction	2.4	0.017	40.0	20.6
apoptosis	2.3	0.033	15.0	5.4

### Microdissection and RNA *in-situ*-hybridization (RISH) revealed keratinocytes as a source of early cutaneous gene expression

Functional clustering and detection of overrepresented transcription factor binding sites both indicated involvement of keratinocytes in the early immune response. This prompted us to analyze more closely the role of the epidermis in early cutaneous gene expression during *L. major* infection *in vivo* by laser microdissection of epidermal keratinocytes from skin 16 hours after infection. For further verification, we applied *in-situ*-hybridization as it additionally allows spatial allocation of gene induction also in other cells outside the epidermis (e.g. infiltrating leukocytes).

**Figure 2:**
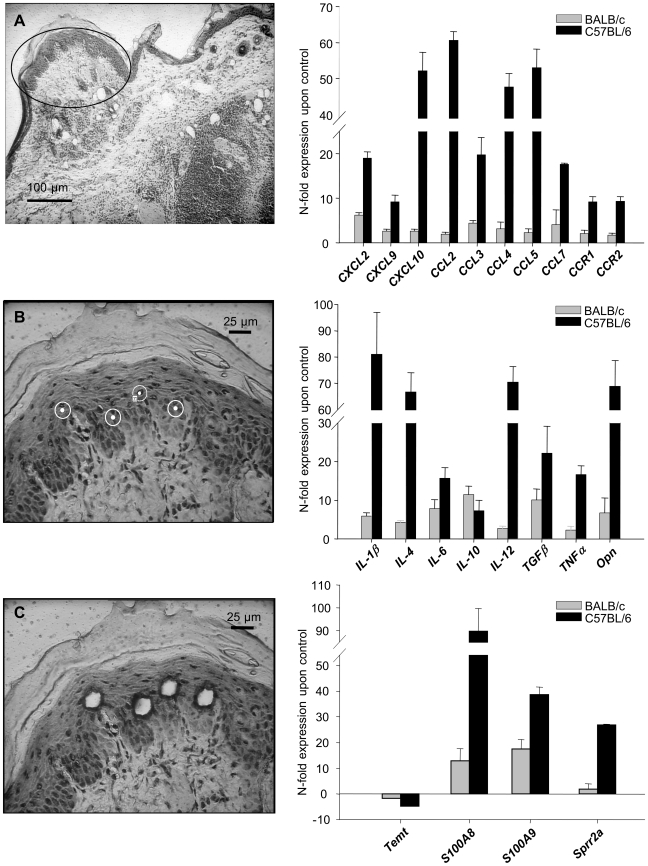
Analysis of gene in keratinocytes isolated from *L. major* infected skin. Laser capture microdissection (left panel). Single keratinocytes were dissected from cryosections of infected and control skin as described in Materials and methods. **A**) Overview, cryosection (BALB/c mice 16 h after infection). **B**,**C**) Single keratinocytes of the stratum spinosum were selected, laser microdissected and pressure catapulted. RT-PCR Analysis of gene expression in microdissected keratinocytes from 16h *L. major* infected skin (right panel). The PCR data were normalized to GAPDH expression and N-fold regulation and SEM in comparison to PBS injected controls was calculated (n = 2). Grey bars: Regulation in BALB/c mice; Black bars: Regulation in C57BL/6 mice.

After laser capturing of microdissected keratinocytes (Figure 2, left panel), we found induction of many immune mediators by RT-PCR, among them IL-4, opn, TNFα, IL-1β, IL-12 and IL-6 (Figure 2, right panel, Table S8). This way we showed that the stronger induction of cytokines applied not only for the whole skin, but in particular also for keratinocytes of resistant C57BL/6 mice.

**Figure 3:**
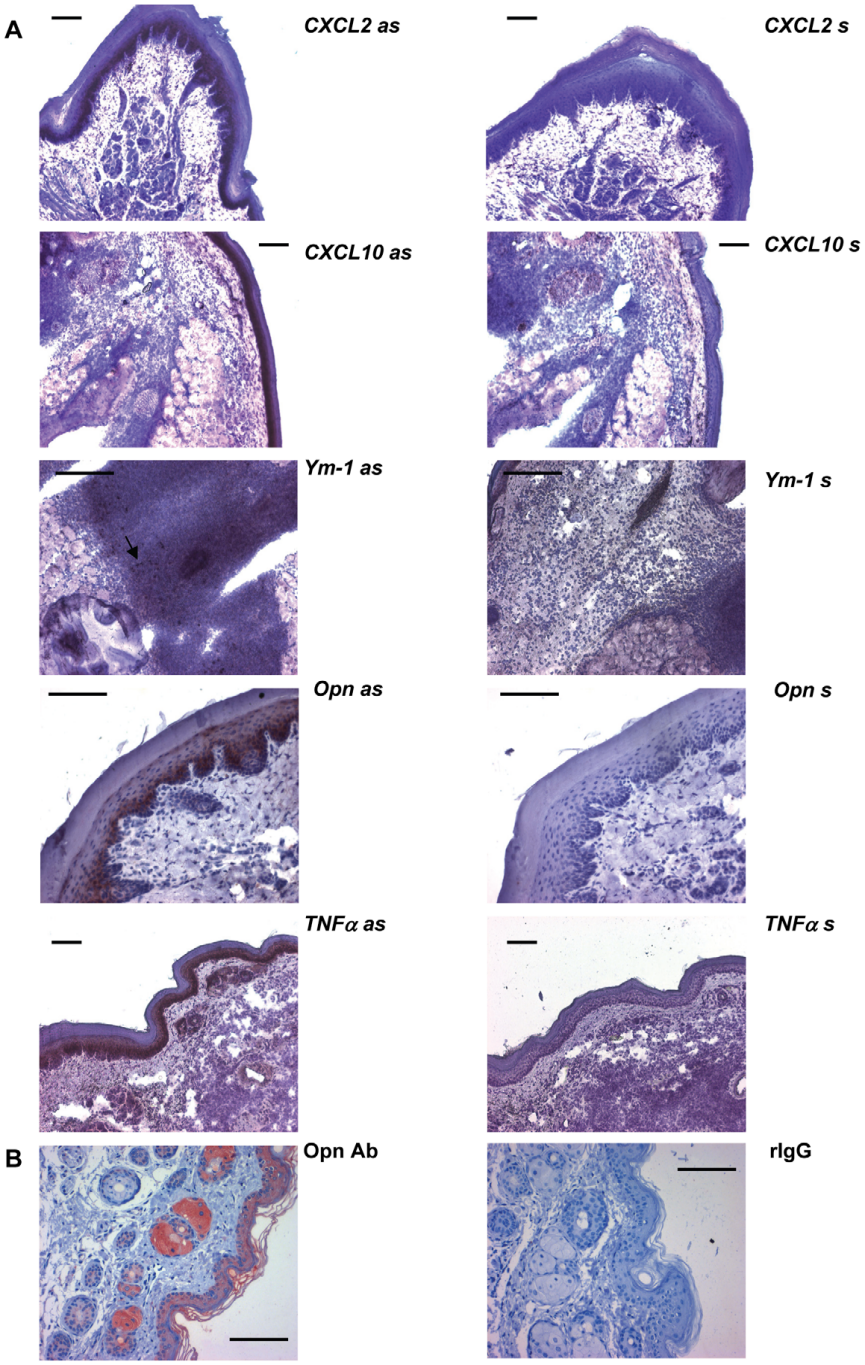
*In-situ*-hybridization and immunohistochemistry. The cellular expression pattern of the indicated genes was analyzed in footpads of C57BL/6 mice infected 16 h with *L. major* by *in-situ*-hybridization (**A**) and immunohistochemistry (**B**). The arrow indicates Ym-1 positive cells in the infiltrate. Bars represent 50 µm; as = antisense RNA probe; s = sense RNA probe; Opn Ab = rat anti mouse monoclonal antibody against opn; rIgG = rat IgG.

Similarly, when we analyzed the cellular cutaneous expression pattern by RISH (Figure 3A) or by immunohistochemistry for opn (Figure 3B) in C57BL/6 mice we found major expression of opn, CXCL10, CXCL2 and TNFα by the epidermis.

These results do not exclude that other cells present in the infected dermis also contribute to the observed induction of gene expression. However, they do reveal that keratinocytes are a hitherto unknown, yet important source of the early and distinctly expressed genes in infected footpads.

### More prominent expression of macrophage chemokines preceded more prominent macrophage infiltration in resistant C57BL/6 mice

Functional clustering had revealed an over-representation of chemotactic factors among the more strongly induced genes in resistant mice. Opn was the chemokine with the most prominent differential expression. It is known as a cytokine able to induce a Th1 response and as a chemoattractant for macrophages, but has not been linked to leishmaniasis yet.

**Figure 4:**
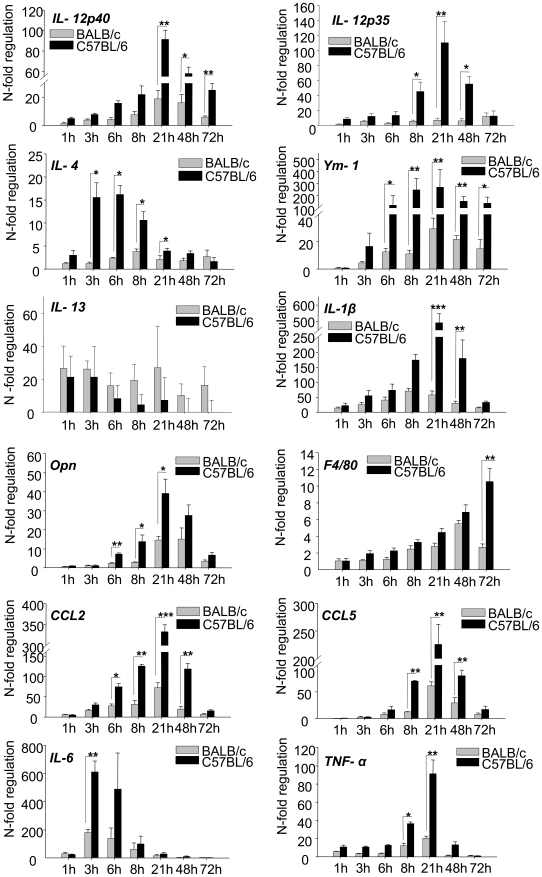
RT-PCR Time course analysis of gene expression in 1h to 72h *L. major* infected skin. The PCR data were normalized to GAPDH expression and mean N-fold regulation and SEM in comparison to PBS injected controls was calculated (n = 3) Grey bars: Regulation in BALB/c mice; Black bars: Regulation in C57BL/6 mice. * = *P*<0.05, ** = *P*<0.01, *** = *P*<0.001 for differences between C57BL/6 and BALB/c mice, Student's t-test.

We therefore investigated, whether epidermal expression of macrophage chemokines (opn, CCL5, CCL2) correlated with a more rapid infiltration of mature macrophages in resistant mice [2]. Time-course experiments with real-time PCR revealed that the stronger peak in expression of macrophage chemokines after 8–24 h of infection in C57BL/6 mice was indeed followed by a stronger expression of Emr1, the gene encoding F4/80, a well established marker for mature macrophages (Figure 4). Thus, our global search for early tissue signals at the site of infection revealed the epidermal expression of chemokines which help to explain the known differences in leukocyte recruitment during experimental leishmaniasis.

### Time course analysis of cytokine expression reveals their relevance for differentiation of Th1 cells

Our experiments had revealed that 16 hours after infection, the epidermis in resistant C57BL/6 mice is a major and stronger source not only for chemokines, but also for cytokines known to have the potential to induce Th1 cells (IL-12, IL-1β, TNFα and opn). Interestingly, keratinocytes in resistant C57BL/6 mice had also shown a higher induction of IL-4 and thus of a cytokine characteristically released by Th2 cells and counteracting Th1 cytokines. However, IL-4 has previously been reported to be necessary for Th1 differentiation and resistance to *L. major* in vivo, but only when administered exclusively during the first 8 hours of infection [13].

This accentuates the relevance of the early crucial time frame for Th1/Th2 differentiation and demonstrates that not only expression, but also duration of expression can be crucial for the effect of cytokines.

When we performed time course analyses for several cytokines, we detected an early peak of cytokine gene expression for IL-4 and IL-6 at 3-6 hours followed by a peak of IL1-β and IL-12 expression at 21 hours which was stronger in resistant mice and declined to baseline levels within one or two days (Figure 4). This temporal expression pattern thus correlated perfectly with the described crucial time frame for Th1/Th2 differentiation. For IL-4 it was particularly consistent with its previously shown pharmacological effects on Th1, but not Th2 cell differentiation. Our results would thus unravel the so far undetected source for IL-4 as a Th1-inducing cytokine *in* vivo. Of note, there were no strain specific differences in the expression of other Th2 cytokines such as IL-13.

We did not succeed in the - notoriously difficult - demonstration of IL-4 protein (due to the often low but yet efficacious concentrations in the tissue). However, as an indirect sign for the biological activity of IL-4 we demonstrated that Ym-1 (Figure 4), a gene specifically regulated by IL-4 and IL-13 in macrophages, was expressed in infiltrating leucocytes (by RISH, Figure 3A) and was more strongly induced in resistant mice following the induction of IL-4 (Figure 4).

There was a striking resemblance between IL-4 and IL-6 with regard to their epidermal and temporal expression pattern (Figure 4). Expression of IL-6 peaked even more rapidly.

### Local deficiency of IL-4 and IL-6 resulted in increased susceptibility towards *L. major* infection

**Figure 5:**
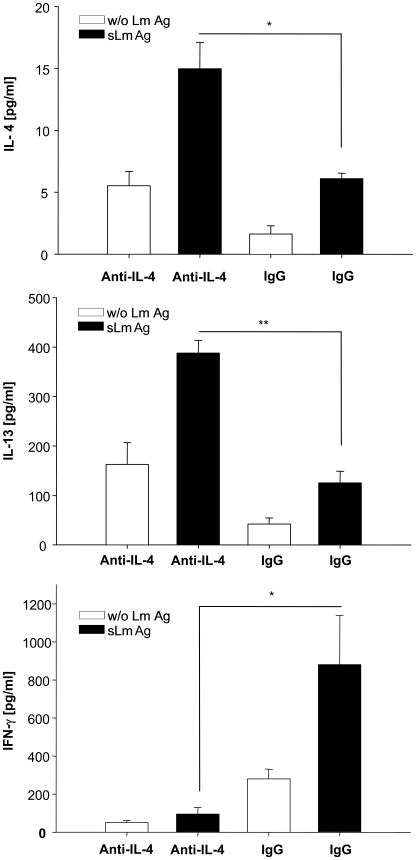
Early anti-IL-4 treatment induces a Th2 switch. IL-4, IL-13 and IFNγ secretion of CD4^+^ cells isolated one week after infection from draining lymph nodes of C57BL/6 mice which had been injected with 1 µg of neutralizing anti-IL-4 or irrelevant antibody into the infected footpad at the time of parasite inoculation and 4h later. Cells were incubated for 48 h with syngenic DC stimulated for 48h with soluble *Leishmania* antigen (sLmAg) (black bars) or with unstimulated syngenic DC (open bars). Cytokines in culture supernatants were assayed by cytometric bead assay (mean ± SE, n = 3) * = p<0.05, ** = p<0.01, Student's t-test.

While it had been demonstrated that early treatment of susceptible mice with IL-4 resulted in a Th1 shift [13], our data now pointed to a corresponding relevance of endogenous IL-4 in resistant mice for induction of the Th1 immune response. To prove this hypothesis we injected 1µg of neutralizing anti-IL-4 antibody in the infected footpads of resistant mice at the time of parasite inoculation and 4h later. When we measured *L. major* specific cytokine secretion by CD4^+^-T-cells one week later we detected increased levels of IL-4 and IL-13 and simultaneously decreased levels of IFNγ (Figure 5). Thus, neutralization of endogenous IL-4 in infected footpads at the time of infection resulted in a clear Th2 switch.

Due to this time course and its likewise dual role in Th1/Th2-differentiation [14]–[18], we hypothesized that IL-6 could act in a similar way as IL-4 and support Th1-immunity during a narrow, early time frame of *L. major* infection. To analyze whether locally produced IL-6 affects adaptive immunity and resistance in *L. major* infection we generated mice with a selective IL-6 deficiency in non-hematopoietic cells (e.g. keratinocytes). To this end we reconstituted lethally irradiated, IL-6-deficient mice with IL-6 competent wild type bone marrow (wt→IL-6^−/−^ chimeric mice). As control group, irradiated C57BL/6 wild type mice were reconstituted with wild type bone marrow (wt→wt mice) and irradiated IL-6^−/−^ mice were reconstituted with wild type bone marrow from IL-6^−/−^ mice (IL-6^−/−^→IL-6^−/−^ chimeric mice).

**Figure 6:**
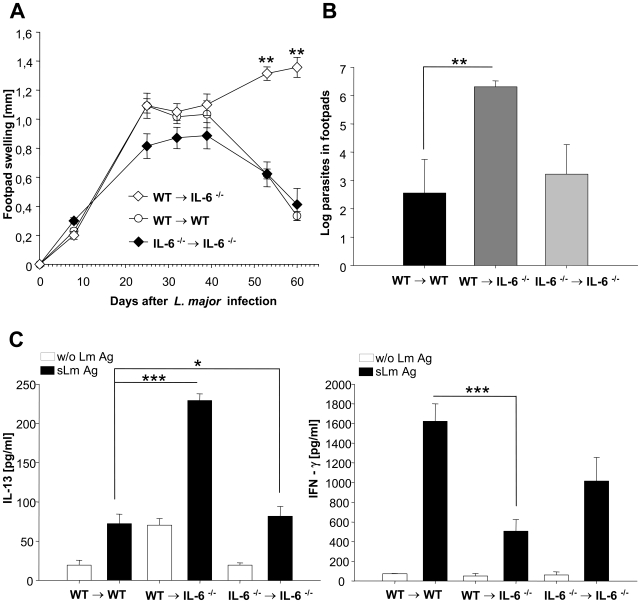
Experimental leishmaniasis in wt→IL-6 −/− chimeric mice. **A**) Footpad swelling (compared to the not infected contra lateral footpad) (mm) of infected wt→IL-6 −/− chimeric mice, control wt→wt mice and control IL-6 −/− →IL-6 −/− chimeric mice ** = p<0.01, Student's t-test. **B**) Limiting dilution assay (LDA) from footpads of wt→IL-6 −/− chimeric mice, control wt→wt mice and control IL-6 −/− →IL-6 −/− chimeric mice 9 weeks after infection (mean */− SE of log parasites) ** = p<0.01, Student's t-test. **C**) IFNγ and IL-13 secretion of CD4^+^ cells isolated from draining lymph nodes of wt→IL-6 −/− chimeric mice, control wt→wt mice and control IL-6 −/− →IL-6 −/− chimeric mice 9 weeks after infection. Cells were incubated for 48 h with syngenic dendritic cells stimulated for 48h with soluble *Leishmania* antigen (s LmAg) (black bars) or with unstimulated syngenic dendritic cells (without (w/o) Lm Ag, open bars). Cytokines in culture supernatants were assayed by cytometric bead assay (mean ± SE, n = 3) * = p<0.05, *** = p<0.001, Student's t-test.

After infection with *L. major*, the deficiency of IL-6 in non-hematopoietic cells and keratinocytes (wt→IL-6^−/−^ chimeric mice) resulted in a marked deterioration of disease (Figure 6A), shown by increased footpad swelling as well as by significantly increased numbers of living parasites in footpads (Figure 6B) and lymph-nodes (data not shown) compared to the control groups of mice. This aggravation correlated with a significant reduction in secretion of IFNγ by *L. major* –specific CD4+ T-cells (restimulated in vitro with *L. major* antigen presented by DC) and a corresponding increase in the antigen-specific secretion of IL13 by *L. major*-specific CD4+ T-cells (Figure 6C).

These data indicate that there is a switch from a Th-1 response towards a Th-2 response in wt→IL-6^−/−^ chimeric mice. The switched Th-cell cytokine pattern proofed to be biologically highly relevant as it directly correlated with the course of disease in wt→IL-6^−/−^ chimeric mice, i.e. a highly significant increase in footpad swelling and a more than 1000 fold higher local parasite titer.

## Discussion

Resistance in experimental *leishmaniasis* depends on the development of a *L. major* specific Th1 response, while Th2 differentiation in BALB/c mice results in susceptibility. The decisive events for the development of a Th1 or Th2 response take place during the first 3 days of infection [9], [19]–[22]. There is growing evidence that the microenvironment of the infected tissue delivers the initial triggers that affect Th-cell differentiation. The nature or source of such triggers, however, is still enigmatic.

Using a combination of gene array analysis, functional clustering, microdissection, RISH and *Real-time* PCR, we were able to confirm the hypothesis that molecules relevant for defense against infection with *L. major* and for the generation of a Th1/Th2 response not only are early induced at the site of infection, but also at great part by the cell-rich epidermis and with a significantly stronger expression in resistant mice. While the relevance of opn, IL-12 IL-1β, and TNFα for the crucial recruitment of macrophages and the induction of a Th1 response are well known [1], [9], [10], [23], the crucial relevance of IL-4 and IL-6 for resistance and Th1 response had to be especially elaborated.

Although an early expression of some chemokines and cytokines in infiltrating leukocytes and in the skin has been found previously [24]–[28], such a pronounced and differential expression of immune-modulatory mediators in the skin within the first hours of *L. major* infection was hitherto not described. Strain specific differences were so far only reported for CXCL10 [24]. Moreover, the major source for cytokine production in the first hours of infection was not known.

The results of functional clustering pointed to a relevant, so far unrevealed involvement of keratinocytes in the early immune response. Our spatial analysis of gene expression by microdissection, *in-situ* hybridization and immunohistochemistry confirmed that subcutaneous injection of *L. major* induces several genes and especially cytokines in keratinocytes during the first hours of infection.

Moreover, detection of proteins in supernatants of minced footpad tissue strongly suggests that induction of genes results in expression and efficient secretion of epidermally produced immune-mediators, which affect the parasite-induced immune response.

The mechanism by which *L. major* induces gene expression in keratinocytes early after infection remains enigmatic. A direct interaction between *L. major* and keratinocytes seems unlikely because of the subcutaneous infection and because keratinocytes do not take up *L. major* in vitro [29]–[32]. It is possible that epidermal gene induction is caused by cytokines (e.g. CCL4, IL-8 and CCL2) released from resident macrophages and early infiltrating granulocytes [2], [24], [33]–[38], after their initial contact with the parasites. By such a crosstalk, epidermal keratinocytes would be involved in an amplification of the *L. major*-related inflammatory tissue signal, since they are capable of synthesizing and secreting large amounts of cytokines [39]. Such an amplification due to high numbers of cells could be essential since only few resident macrophages are present early after infection and infiltrating granulocytes are not equipped for sustained transcription of inflammatory mediators.

Most importantly, the induction of gene expression in keratinocytes was significantly different between susceptible and resistant mice, indicating an influence of epidermal cells on the direction of the ensuing immune response.

One mechanism by which this influence is executed could be the differential recruitment of leukocytes. A higher percentage of granulocytes in BALB/c mice is relevant for susceptibility, while a higher percentage of mature macrophages in C57BL/6 mice is associated with resistance towards *L. major* [2], [3], [40]. Here we demonstrate the temporal correlation between a significantly stronger and transient expression of macrophage chemoattractants (CCL5, CCL2 and opn) with a peak after 8h post infection and an ensuing significantly more pronounced macrophage infiltration 3 days after infection.

Opn was also described as an early Th1-inducing cytokine acting on DC [10], [41]. Together with the significantly stronger expression of the Th1-inducing cytokines IL-12, TNFα and IL-1β in the epidermis of resistant mice, this points to a direct effect of epidermal gene expression on Th1/2 differentiation. IL-12 is the best characterized Th1-inducing cytokine so far [19]–[21], although a stronger early expression of IL-12 in vivo in resistant mice has so far never been demonstrated, neither for the skin nor for the lymph nodes. Similarly, IL-1β has so far not been shown to be differentially expressed in skin, whereas we had previously described a more prominent expression of IL-1α in lymph nodes of resistant mice [9].

The time course of transient IL-12 and IL-1β expression from 8 h to 72 h after infection correlates perfectly with the critical time frame for Th1/Th2 differentiation [9], [19]–[21]. In confirmation of this relevance, we and others previously showed that application of either IL-12 or IL-1β at the site of infection during the first three days after injection with *L. major* was able to promote Th1 differentiation and to inhibit disease progression in BALB/c mice [9], [42]. This study now shows that keratinocytes are an essential natural source to provide early significant amounts of IL-12 or IL-1β in C57BL/6 mice.

In addition, our finding of a transient IL-4 expression in the skin of resistant mice may explain a paradoxon in experimental leishmaniasis: IL-4 on one hand is the best characterized Th2 cytokine; correspondingly, IL-4 production in the draining lymph nodes of BALB/c has been associated with induction of a Th2 response [43]–[45]. On the other hand, IL-4 is able to induce production of IL-12p70 in murine and human DC in vitro [46], [47] and to promote a protective Th1 immune response in susceptible BALB/c mice but only when administered during the first 8 h of infection, and not if given for a prolonged period of time [13]. Thus, IL-4 induces a Th1 response earlier in infection, most likely via increasing IL-12 secretion from DCs in a defined early time frame. However, it remained unclear whether endogenous IL-4 is involved in Th1 immunity.

We now demonstrated that the epidermis could provide IL-4 whose expression is temporally restricted to exactly the crucial early time frame. While it is technically problematic to detect the low local levels of IL-4 protein, we found that expression of Ym-1, a gene specifically regulated by IL-4 in macrophages (and IL-13 which did not shown differential expression), exactly followed the differential IL-4 expression with a stronger peak in C57BL/6 mice. This could indicate a temporally defined and quantitatively distinct local presence of biological active IL-4. More important, we could clearly demonstrate that neutralizing early endogenous IL-4 in resistant mice locally by anti-IL-4 antibody resulted in a switch to a Th2 cytokine secretion pattern of CD4^+^ T-cells from draining lymph nodes one week later (Figure 5). This clearly indicates that early endogenous IL-4, most likely produced by keratinocytes, is mandatory for the induction of a Th1 response against *L. major*.

Since IL-4 is induced in the epidermis - and thus in direct vicinity to DCs -, we suggest that under physiological conditions epidermal IL-4 could act in a paracrine way on DCs so that they are prepared to release IL-12 when migrating to the lymph node.

Similar to IL-4 we found a very rapid, but also temporally restricted induction of IL-6 (mRNA as well as protein) in the skin which likewise was significantly stronger in resistant mice. Microdissection revealed that keratinocytes are an important source of local IL-6 expression. Like IL-4, IL-6 has been demonstrated to induce Th2 differentiation via direct action on Th cells [14], [15], while it was also required for the development of Th1 immunity in murine tuberculosis, colitis and experimental autoimmune encephalomyelitis [16]–[18]. Besides, IL-6 appears to be involved in differentiation of Th17 cells and in inhibition of regulatory T-cells [48] while it also inhibited differentiation, maturation and activation of DC [49]–[52]. In the light of these results it is reasonable to conclude that the effect of IL-6 on either Th1 or Th2 differentiation in an emerging immune response crucially depends on timing as well as site of action. In case of its complete genetic deletion, these effects may set each other off, which may explain why it resulted in only minimal net effects on the course of experimental leishmaniasis [53], [54].

In order to assess whether lack of cutaneous IL-6 production would impair development of Th1 cells, we demonstrated that IL-6 −/− mice with IL-6 competent bone marrow and thus with a constitutional lack of IL-6 at the site of infection, became markedly more susceptible to *L. major* (more than 1000 fold more parasites compared to control animals) and showed a shift from a Th1 towards a Th2 response. Thus, IL-6 is a new tissue signal whose early local presence promotes Th1 cells.

The mechanism by which IL-6 affects the Th1 response is currently unclear. It may not primarily instruct DC for Th1 priming [49]–[52], but could inhibit conversion of naïve T-cells into Foxp3+ regulatory T-cells (Treg) [48] so that its local absence leads to increased influence of Treg on Th1 cells. However, the function of Treg in experimental leishmaniasis is not completely clear and both inhibitory as well as exaggerating effects of Treg on the course of *L major* infection have been described [55]–[59]. Thus, it is also tempting to speculate that the immediate induction of IL-6 after contact with the parasites may prompt resident and early infiltrating cells to generate a Th1 promoting local micromilieu, characterized e.g. by ensuing expression of IL-4, IL1β, IL-12 and opn.

In summary, our approach directed at a global view of gene expression and thus reflecting the biology of systems revealed a stronger expression of immunomodulatory mediators in the infected skin and epidermis of resistant compared to susceptible mice. Our approach for the first time defines the global pattern of the early “tissue signal” and moreover identifies keratinocytes as a critical modulator of the microenvironment in *L. major* infected skin. Furthermore, our data indicate for the first time that epidermal cytokine expression, e. g. of IL-4 and IL-6 is a decisive factor in the generation of protective Th1 immunity and contributes actively to the outcome of inflammatory reactions.

## Materials and Methods

### Ethics statement

All experiments with mice were performed with the approval of the State Review Board of Nordrhein-Westfalen (Germany) according to the German law for animal welfare (Tierschutzgesetz) § 8; reference number 8.87–50.10.36.08.009.

### Mice

Specific pathogen-free, female C57BL/6 and BALB/c mice were purchased from Charles River, Germany, and were housed in microisolator cages and given mouse chow and water ad libitum. Mice were 8–12 wk of age when used in experiments. All experiments with mice were performed with the approval of the State Review Board of Munster (Germany).

### Parasites and experimental infection

*L. major* (MHOM/IL/81/FE/BNI) parasites were cultivated in Schneider's Drosophila Medium supplemented with 10% FCS, 2% human urine, 2% glutamine, and 1% Penicillin/Streptomycin. Soluble *Leishmania* antigen (SLA) was prepared by 5 repeated freeze and thaw cycles in phosphate buffered saline (PBS).

Mice were infected subcutaneously by application of 2×10^7^ promastigotes (stationary phase) of *L. major* in 50 µl PBS into the left hind footpad. The right footpad was injected with 50 µl PBS alone and served as internal control to ensure that gene expression was not caused by the injection stimulus. After sacrifice, footpads from mice were harvested 1 to 72 hours after infection for gene expression analysis. In the experiments neutralizing local IL-4 1 µg of neutralizing rat anti-mouse IL-4-antibody or irrelevant rat IgG (Biolegend, Uithoorn, Netherlands) was used. Mice were injected at the time of parasite inoculation and 4h later.

### DNA microarray and statistical data analysis

In three independent experiments, total RNA from 16 h *L. major* infected C57BL/6 or BALB/c mice and PBS injected control animals was isolated and subsequently processed for microarray hybridization using Affymetrix Murine Genome MG_U74Av2 arrays according to the manufacturer's instructions (Affymetrix). Microarray data were analyzed using MicroArray Suite Software 5.0 (Affymetrix) using data from corresponding control samples as baseline and further studied applying the Expressionist Suite software package (GeneData), which allows identification of genes that are significantly regulated in multiple independent experiments as described previously [60].

We retained only genes which were significantly regulated in every single experiment (change p-value <0.05, fold-change ≥2, expression over background) as well as in the complete set of experiments (fold-change of ≥3.0, *p*-value of <0.05, paired t-test).

To compare *L. major* induced alterations of cutaneous expression patterns between resistant and susceptible mouse strains, signal log ratios of infected versus uninfected control samples in both mice strains were evaluate by paired t-test. We retained only genes with a p-value <0.05 and a differential fold-change regulation of ≥1.75.

Principal component analysis (PCA) was applied to mathematically reduce the dimensionality of the entire spectrum of gene expression values of a microarray experiment to three components as described previously [61] and revealed that individual experimental groups were well reproducible and clearly separated.

### Functional clustering

To analyze the microarray data in the context of biological functions, we used information available from the Gene Ontology (GO) consortium (http://www.geneontology.org) [11], [12]. The GO terms represent a defined vocabulary describing the biological process, cellular components, and molecular functions of genes in a hierarchical directed acyclic graph structure. Statistical analysis was performed for groups of >10 genes using GenMAPP software [11], [12]. For each of the existing GO terms, the cumulative number of genes meeting our criteria (e.g. up- or down-regulated) and of all genes represented on the microarray was calculated. The Z score is calculated for every GO term by subtracting the expected number of genes meeting the criterion from the actual number, and division of this value by the standard deviation of the actual number of genes:
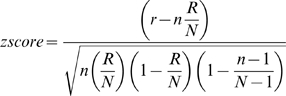
with N as the total number of genes measured, R as the total number of genes meeting the criterion, n as the total number of genes in the specific GO term, and r as the number of genes meeting the criterion in the specific GO term. A positive Z score indicates that there are more genes meeting the criterion in the specific GO term than expected by chance. The Z-score is transferred to p-values under the assumption of a hypergeometric distribution.

### RNA in situ hybridization (RISH) and immunohistochemistry

For RISH, the complete coding regions of the genes analyzed were amplified by PCR and cloned into pBluecript (Stratagene, La Jolla, CA) (Table S9). For antisense and sense probes, the plasmid was linearized by appropriate restriction enzymes and T7 or T3 RNA polymerase was used to synthesize digoxigenin labeled probes. The RISH was performed on 4 µm frozen sections according to an protocol described earlier [62].

Immunohistochemical staining was performed [2] using a rabbit anti-mouse opn antibody (Assay Designs, Ann Arbor, MI, USA).

### Preparation and staining of slides for laser microdissection and pressure catapulting

Tissue-Tec O.C.T. (Sakura, Germany) -embedded hind feet were cut in 12 µm sections on a microtome, transferred onto PEN-covered glass slides (PALM, Germany) and immediately stored at −80°C. Sections were stained with cresyl violet (1% in aqua bidest) followed by rehydration in 100, 96, 70% ethanol and dried at 37°C for half an hour.

### Laser microbeam microdissection & pressure catapulting (LMPC)

Collection of keratinocytes by LMPC was performed as described [63], utilizing the PALM Laser Microbeam Microdissection System with Laser Pressure Catapulting and Robocut Software (PALM, Germany). A minimum of 200 cell equivalents (about 1000 visible cells) were isolated and collected into a reaction tube cap, which was filled with TRIzol Reagent (Invitrogen, Germany).

### RNA Isolation, reverse transcription and random based PCR amplification

For total skin RNA extraction, skin from the hind footpad was excised and pulverized in liquid nitrogen and Potter homogenized in TRIzol Reagent. Subsequent RNA isolation was performed using TRIzol Reagent (Invitrogen, Germany) and subsequently RNeasy Minikit (Qiagen, Germany) cleanup preparation including DNAse digestion. The RNA from laser microdissected keratinocytes was TRIzol extracted, following the manufacturer's protocol for small quantities of cells. The quality and approximate quantity of the resulting RNA was determined using the microfluidics system (Agilent 2100 Bioanalyzer, Agilent Technologies). Whole skin material was transcribed into cDNA utilizing H-minus reverse transcriptase (MBI Fermentas, Germany). cDNA synthesis and amplification of laser microdissected samples were performed with the Microarray Target Amplification Kit (Roche Diagnostics, Germany), following the manufacturer's instructions. Total RNA extracted from 1000 keratinocytes was used as template for first-strand synthesis.

### Quantitative RT-PCR

Expression of selected genes was confirmed by real-time RT-PCR as described previously [60], see Table S10 for primer sequence information.

### Protein measurements

To determine secreted proteins in infected footpad tissue, dissected feet were flushed repeatedly with a total of 500 µl PBS and adjusted to equal protein concentrations. Cytokine concentrations were determined using the cytometric bead assay (BD Bioscience, San Jose, CA, USA).

Osteopontin was immunoprecipitated using monoclonal goat anti-opn antibody (RD Systems, Minneapolis, MN, USA), following Protein A sepharose, elution of protein and subsequent analysis using polyacrylamid gel electrophoresis and western blotting.

Samples were adjusted to equal protein concentrations and separated by SDS/PAGE (12% acrylamide). Subsequently, proteins were transferred by blotting onto membrane (Pall GmbH, Dreieich, Germany), incubated for 1 h in NaCl/Tris/0.1% (v/v) Tween 20/5% (w/v) nonfat dried milk, and then for 1 h in NaCl/Tris/5% (w/v) nonfat dried milk containing goat anti-opn antibody. The membrane was washed three times in NaCl/Tris/0.1% Tween-20 and incubated with a phosphatase-conjugated secondary anti-goat-IgG to visualize opn using the standard phosphatase reaction. The bands were scanned densitometrically.

### Generation of chimeric mice

C57BL/6 mice or IL-6 gene deficient mice were lethally irradiated (5.0 Gy on 2 days resulting in a cumulative dose of 10 Gy) and reconstituted with bone marrow cells (10^7^ cells/mouse) obtained from wt C57BL/6 or from IL6−/− mice. For experimental leishmaniasis chimeric mice were used 6 weeks after bone marrow reconstitution.

### Experimental leishmaniasis

Cutaneous leishmaniasis was initiated by subcutaneous application of 2×10^7^ promastigotes (stationary phase) of *L. major* in 50 µl PBS into the left hind footpad. Footpad thickness was assessed weekly using a metric caliper. Specific swelling of the infected footpad was assessed by subtracting the diameter of the infected footpad from that of the non-infected footpad. Footpads, lymph nodes and spleen from each mice were harvested for limiting dilution assay (LDA) and determination of cytokine profile. The experiments were repeated 2–3 times.

### Limiting dilution assay

Parasite numbers in bone marrow and liver as a parameter for systemic spread were determined 8–12 weeks after infection by a limiting dilution assay (LDA) modified by using leishmania medium as specified above instead of slant blood agar [64].

### Cytokine and proliferation assay

For cytokine assay mice were euthanized and draining lymph nodes were aseptically removed. A single cell suspension was prepared and CD4^+^ T cells were collected using biomagnetic enrichment procedures (Miltenyi Biotec, Bergisch Gladbach, Germany) according to the manufacturer's recommendations.

Bone marrow derived dendritic cells (DC) were generated as previously described [65]. Shortly, the femur bone was aseptically removed from euthanized C57BL/6 mice and the bone marrow was flushed out. Bone marrow DCs were expanded with IL-4 and GM-CSF for 6 days. DCs (1×10^6^ cells/ml) were incubated with SLA equivalent to 5×10^6^ *L. major* for 48 h. For assessment of cytokine secretion DC and CD4^+^ T cells (5×10^4^/100 µl) were mixed in a ratio 1∶5 and cultured in RPMI1640 plus 2 mM glutamine, 50 µM mercaptoethanol and 10% FCS for 48 hours. Culture supernatants were assayed by cytometric bead assay BD Bioscience, San Jose, CA, USA) according to the manufacturer's instructions.

### Statistical analysis

To determine whether differences were statistically significant, Student's t test was performed, using a two-tailed distribution. Indication of p-values are as follows * <0.05; ** <0.01; *** <0.001.

### Annotation of genes and proteins mentioned in the manuscript (database NCBI Gene)

CCL2 GeneID: 20296, CCL3 GeneID: 20302, CCL4 GeneID: 20303, CCL5 GeneID: 20304, CCL7 GeneID: 20306, CCL9 GeneID: 20308, CCR1 GeneID: 12768, CCR2 GeneID: 12772, CCR5 GeneID: 12774, CD14 GeneID: 12475, Chi3l3 GeneID: 12655, CXCL10 GeneID: 15945, CXCL1 GeneID: 14825, CXCL2 GeneID: 20310, CXCL9 GeneID: 17329, Emr1 GeneID: 13733, Ifi202b GeneID: 26388, IFN-g GeneID: 15978, IL-10 GeneID: 16153, IL-12p35 GeneID: 16159, IL-12p40 GeneID: 16160, IL-13 GeneID: 16163, IL-1a GeneID: 16175, IL-1b GeneID: 16176, IL-4 GeneID: 16189, IL-6 GeneID: 16193, Ost GeneID: 20750, Ptx3 GeneID: 19288, S100a8 GeneID: 20201, S100a9 GeneID: 20202, Saa3 GeneID: 20210, Slpi GeneID: 20568, Sprr2a GeneID: 20755, Sprr2B GeneID: 20756, Sprr2h GeneID: 20756, Temt GeneID: 21743, Tgfb1 GeneID: 21803, TNFa GeneID: 21926, Tnfrsf1b GeneID: 21938.

## Supporting Information

Table S1 Complete list of genes regulated in BALB/c mice
(0.02 MB PDF)

Table S2 Genes regulated in C57BL/6 mice
(0.03 MB PDF)

Table S3 Genes which were stronger regulated in BALB/c mice
(0.01 MB PDF)

Table S4 Genes which were stronger regulated in C57BL/6 mice
(0.02 MB PDF)

Table S5 Absolute expression levels of genes analyzed by *Real-time* PCR in uninfected skin of BALB/c and C57BL/6 mice (expressed as copy number/10000 copies of glyceraldehyde-3-phosphate dehydrogenase (GAPDH))
(0.01 MB PDF)

Table S6 Functional clusters overrepresented in genes regulated in C57BL/6 mice
(0.01 MB PDF)

Table S7 Functional clusters overrepresented in genes regulated in Balb/c mice
(0.01 MB PDF)

Table S8 Absolute expression levels of genes analyzed by *Real-time* PCR in keratinocytes isolated from uninfected skin of BALB/c and C57BL/6 mice (expressed as copy number/10000 copies of glyceraldehyde-3-phosphate dehydrogenase (GAPDH))
(0.01 MB PDF)

Table S9 cDNA sequences used as templates for RISH
(0.01 MB PDF)

Table S10 Primers used for Real-time PCR
(0.06 MB PDF)
