# Supplementary material for: Keratinocytes Determine Th1 Immunity during Early Experimental Leishmaniasis
Source: PLoS Pathog. 2010 Apr 29;6(4):e1000871. doi: 10.1371/journal.ppat.1000871 (PMC2861693; doi:10.1371/journal.ppat.1000871)
Supplement: Table S1 — Complete list of genes regulated in BALB/c mice (0.02 MB PDF) [file ppat.1000871.s001.pdf]

Table S1. Genes regulated in BALB/c mice

MW N-fold = mean regulation compared to uninfected control, TTEST = p-value for regulation in BALB/c (BALB) or C57BL/6 (C57) mice

Gene Symbol	Gene Title	MW N-fold BALB	MW N-fold C57	TTEST BALB	TTEST C57
	**Genes Up-regulated**				
Cxcl10	chemokine (C-X-C motif) ligand 10	34.5	125.7	0.0015	0.0022
Isg20	interferon-stimulated protein	31.8	35.1	0.0016	<0.001
Fcgr1	Fc receptor, IgG, high affinity I	30.9	83.6	0.0012	<0.001
Mx1	myxovirus (influenza virus) resistance 1	30.4	26.7	<0.001	<0.001
G1p2	interferon, alpha-inducible protein	27.8	111.2	0.0014	0.0011
Ifit2	interferon-induced protein with tetratricopeptide repeats 2	25.5	29.9	<0.001	<0.001
Irg1	immunoresponsive gene 1	24.8	77.8	<0.001	<0.001
Slfn3	schlafen 3	22.5	42.8	<0.001	<0.001
Vig1-pending	viral hemorrhagic septicemia virus(VHSV) induced gene 1	22.5	47.7	<0.001	<0.001
Ifit1	interferon-induced protein with tetratricopeptide repeats 1	20.0	29.7	<0.001	<0.001
Ifi47	interferon gamma inducible protein	19.6	21.9	<0.001	<0.001
Tyki	thymidylate kinase family LPS-inducible member	18.7	17.4	<0.001	<0.001
Ccl4	chemokine (C-C motif) ligand 4	18.7	34.6	<0.001	<0.001
Tgtp	T-cell specific GTPase	18.6	10.1	<0.001	0.0017
Slfn4	schlafen 4	18.4	52.2	<0.001	<0.001
Ifit3	interferon-induced protein with tetratricopeptide repeats 3	18.2	25.4	<0.001	<0.001
Gbp1	guanylate nucleotide binding protein 1	18.0	0.6	<0.001	0.3828
Csf3r	colony stimulating factor 3 receptor (granulocyte)	16.7	20.7	<0.001	<0.001
Klra2	killer cell lectin-like receptor, subfamily A, member 2	16.5	4.5	<0.001	0.0416
Usp18	ubiquitin specific protease 18	16.0	37.8	<0.001	0.0019
Il6	interleukin 6	15.7	15.2	0.0014	0.0257
Ccl3	chemokine (C-C motif) ligand 3	15.0	28.1	0.0013	<0.001
Kdap	kidney-derived aspartic protease-like protein	14.5	24.5	<0.001	<0.001
Clecsf8	C-type (calcium dependent, carbohydrate recognition domain) lectin, superfamily member 8	14.1	40.7	<0.001	<0.001
Plac8	placenta-specific 8	14.0	30.5	<0.001	<0.001
Nmi	N-myc (and STAT) interactor	13.0	8.0	0.0369	0.0011
Cxcl2	chemokine (C-X-C motif) ligand 2	12.2	23.9	<0.001	<0.001
Ccl7	chemokine (C-C motif) ligand 7	12.0	16.2	<0.001	<0.001
Ccl5	chemokine (C-C motif) ligand 5	11.9	13.9	0.0014	<0.001
Mad	Max dimerization protein	11.6	2.8	0.0177	<0.001
Adam8	a disintegrin and metalloprotease domain 8	11.1	23.1	<0.001	<0.001
Fmnl	formin-like	10.7	38.9	0.0055	<0.001
Irf7	interferon regulatory factor 7	10.5	15.7	<0.001	<0.001
Trim30	tripartite motif protein 30	10.5	11.9	<0.001	<0.001
Iigp-pending	interferon-inducible GTPase	10.1	6.1	<0.001	0.0065
Il1b	interleukin 1 beta	9.8	39.2	0.0032	<0.001
Gbp2	guanylate nucleotide binding protein 2	9.6	12.4	<0.001	<0.001
Cxcl5	chemokine (C-X-C motif) ligand 5	9.3	18.3	<0.001	<0.001
Csprs	component of Sp100-rs	9.3	5.4	0.0245	0.1426
Fpr-rs2	formyl peptide receptor, related sequence 2	9.2	12.0	<0.001	<0.001
Il1rn	interleukin 1 receptor antagonist	8.8	8.9	<0.001	<0.001
Slfn2	schlafen 2	8.7	12.8	<0.001	<0.001
Ccl2	chemokine (C-C motif) ligand 2	8.7	12.0	<0.001	<0.001
Sell	selectin, lymphocyte	8.4	12.3	<0.001	<0.001

Gp49a	glycoprotein 49 A	8.4	13.4	<0.001	<0.001
S100a9	S100 calcium binding protein A9 (calgranulin B)	8.3	24.6	0.0054	<0.001
5830484 A20Rik	RIKEN cDNA 5830484A20 gene	8.2	5.8	<0.001	<0.001
Ccr5	chemokine (C-C motif) receptor 5	8.1	142.5	0.0024	<0.001
Cxcl9	chemokine (C-X-C motif) ligand 9	8.0	4.8	0.0291	0.0073
Ms4a4c	membrane-spanning 4-domains, subfamily A, member 4C	7.8	11.4	<0.001	<0.001
Ifi204	interferon activated gene 204	7.7	13.1	<0.001	<0.001
Ms4a6b	membrane-spanning 4-domains, subfamily A, member 6B	7.7	42.8	<0.001	0.0017
Gbp3	guanylate nucleotide binding protein 3	7.7	6.4	<0.001	0.0010
Psmb9	proteosome (prosome, macropain) subunit, beta type 9 (large multifunctional protease 2)	7.4	15.2	<0.001	<0.001
Basp1	brain abundant, membrane attached signal protein 1	7.3	15.3	<0.001	<0.001
Saa3	serum amyloid A 3	7.2	28.1	0.0070	<0.001
Hdc	histidine decarboxylase	7.1	9.0	0.0017	<0.001
Krt1-16	keratin complex 1, acidic, gene 16	7.0	5.9	<0.001	0.0030
Ifi205	interferon activated gene 205	7.0	13.1	<0.001	<0.001
Igtp	interferon gamma induced GTPase	6.9	4.4	<0.001	0.0017
A33007 5D07	hypothetical protein A330075D07	6.9	10.5	<0.001	<0.001
H2-T24	histocompatibility 2, T region locus 24	6.8	12.0	<0.001	<0.001
9130009 C22Rik	RIKEN cDNA 9130009C22 gene	6.6	7.0	<0.001	<0.001
F10	coagulation factor X	6.6	37.0	0.0027	0.0021
Rgl1	ral guanine nucleotide dissociation stimulator,-like 1	6.5	11.5	0.0108	<0.001
Plscr2	phospholipid scramblase 2	6.5	4.8	0.0123	0.0647
Ifi1	interferon inducible protein 1	6.2	5.9	<0.001	<0.001
Bid	BH3 interacting domain death agonist	6.2	2.2	0.0374	0.0376
Cxcr4	chemokine (C-X-C motif) receptor 4	6.1	10.6	0.0022	0.0012
Ccr1	chemokine (C-C motif) receptor 1	6.1	11.6	<0.001	<0.001
Slfn1	schlafen 1	6.1	9.0	<0.001	<0.001
Samhd1	SAM domain and HD domain, 1	6.0	6.9	<0.001	<0.001
Ptx3	pentaxin related gene	5.8	3.2	0.0098	0.0026
S100a8	S100 calcium binding protein A8 (calgranulin A)	5.7	18.8	0.0022	<0.001
Gtpi-pending	interferon-g induced GTPase	5.6	3.8	<0.001	<0.001
Stat1	signal transducer and activator of transcription 1	5.5	5.8	<0.001	<0.001
Lcn2	lipocalin 2	5.4	10.8	<0.001	<0.001
Ogfr	opioid growth factor receptor	5.4	3.5	0.0035	<0.001
Prg	proteoglycan, secretory granule	5.4	8.6	<0.001	<0.001
Ifi202b	interferon activated gene 202B	5.4	-1.6	<0.001	0.0716
Ifi203	interferon activated gene 203	5.3	11.3	<0.001	<0.001
Mx2	myxovirus (influenza virus) resistance 2	5.2	3.6	<0.001	<0.001
Hck	hemopoietic cell kinase	5.2	8.9	<0.001	<0.001
Ccl12	chemokine (C-C motif) ligand 12	5.2	7.5	<0.001	0.0062
Crap	cardiac responsive adriamycin protein	5.1	2.6	0.0518	0.1047
Psmb8	proteosome (prosome, macropain) subunit, beta type 8 (large multifunctional protease 7)	4.9	6.0	<0.001	<0.001
Timp1	tissue inhibitor of metalloproteinase 1	4.9	7.5	<0.001	<0.001
Krt2-5	keratin complex 2, basic, gene 5	4.8	7.1	<0.001	<0.001
Pdk4	pyruvate dehydrogenase kinase, isoenzyme 4	4.8	3.3	0.0188	0.0943
Spp1	secreted phosphoprotein 1	4.8	28.3	<0.001	<0.001
Cd53	CD53 antigen	4.7	10.4	0.0036	<0.001
Msr1	macrophage scavenger receptor 1	4.7	4.1	0.0051	0.0010
Mmp3	matrix metalloproteinase 3	4.7	8.0	<0.001	<0.001
Tap1	transporter 1, ATP-binding cassette, sub-family B	4.6	5.4	<0.001	<0.001

	(MDR/TAP)				
Ccrl2	chemokine (C-C motif) receptor-like 2	4.6	9.3	0.0020	0.0358
Mlp	MARCKS-like protein	4.5	5.4	<0.001	<0.001
Rrad	Ras-related associated with diabetes	4.5	4.0	0.0011	0.0154
Ninj1	ninjurin 1	4.3	4.8	0.0138	<0.001
Mpeg1	macrophage expressed gene 1	4.2	12.3	0.0018	<0.001
2010008 K16Rik	RIKEN cDNA 2010008K16 gene	4.2	4.7	<0.001	<0.001
Itgb2	integrin beta 2	4.2	9.1	<0.001	<0.001
Vav1	vav 1 oncogene	4.1	8.6	<0.001	0.0046
Gla	galactosidase, alpha	4.1	3.2	0.0072	0.0054
Ptprc	protein tyrosine phosphatase, receptor type, C	4.0	6.2	0.0054	0.0042
Lgals9	lectin, galactose binding, soluble 9	4.0	4.3	<0.001	<0.001
Lst1	leukocyte specific transcript 1	3.9	4.0	<0.001	<0.001
Gch	GTP cyclohydrolase 1	3.9	2.3	0.0013	0.0070
Ltb	lymphotoxin B	3.8	3.1	0.0016	<0.001
Cd52	CD52 antigen	3.8	8.5	<0.001	<0.001
Pnp	purine-nucleoside phosphorylase	3.8	3.4	<0.001	<0.001
Icam1	intercellular adhesion molecule	3.8	3.1	0.0035	0.0031
Ms4a4d	membrane-spanning 4-domains, subfamily A, member 4D	3.8	3.8	<0.001	<0.001
Ptafr	platelet-activating factor receptor	3.7	3.7	<0.001	0.0158
Tyrobp	TYRO protein tyrosine kinase binding protein	3.7	10.0	0.0019	<0.001
Il15	interleukin 15	3.7	9.6	0.0225	0.0350
Apobec1	apolipoprotein B editing complex 1	3.7	3.9	<0.001	0.0010
Cxcl1	chemokine (C-X-C motif) ligand 1	3.7	4.0	0.0031	0.0271
Tnfrsf1b	tumor necrosis factor receptor superfamily, member 1b	3.7	7.8	<0.001	<0.001
Socs3	suppressor of cytokine signaling 3	3.7	3.8	<0.001	0.0069
Sema4d	sema domain, immunoglobulin domain (Ig), transmembrane domain (TM) and short cytoplasmic domain, (semaphorin) 4D	3.6	4.9	<0.001	<0.001
Ncf4	neutrophil cytosolic factor 4	3.6	11.1	0.0018	<0.001
Krt2-6a	keratin complex 2, basic, gene 6a	3.6	3.5	0.0028	<0.001
Irf1	interferon regulatory factor 1	3.6	3.5	<0.001	<0.001
Sprr2a	small proline-rich protein 2A	3.5	7.3	<0.001	<0.001
Dck	deoxycytidine kinase	3.5	4.3	0.0803	0.0023
Tap2	transporter 2, ATP-binding cassette, sub-family B (MDR/TAP)	3.4	3.2	<0.001	0.0013
1300004 C08Rik	RIKEN cDNA 1300004C08 gene	3.4	3.1	<0.001	<0.001
Pla2g7	phospholipase A2, group VII (platelet-activating factor acetylhydrolase, plasma)	3.4	4.7	0.0080	<0.001
Ppicap	peptidylprolyl isomerase C-associated protein	3.3	4.3	<0.001	<0.001
Pira1	paired-Ig-like receptor A1	3.3	4.3	0.0021	<0.001
Lyn	Yamaguchi sarcoma viral (v-yes-1) oncogene homolog	3.2	3.8	0.0014	<0.001
Psmb10	proteasome (prosome, macropain) subunit, beta type 10	3.1	3.0	0.0016	0.0017
Arg2	arginase type II	3.1	2.3	<0.001	0.0017
Prkr	protein kinase, interferon-inducible double stranded RNA dependent	3.1	2.9	<0.001	<0.001
Adar	adenosine deaminase, RNA-specific	3.1	2.7	<0.001	<0.001
Fln29-pending	FLN29 gene product	3.1	2.5	<0.001	<0.001
Ly6e	lymphocyte antigen 6 complex, locus E	3.1	2.6	<0.001	<0.001
Lcp2	lymphocyte cytosolic protein 2	3.1	4.3	<0.001	<0.001
Saa2	serum amyloid A 2	3.0	6.0	<0.001	<0.001
Gp38	glycoprotein 38	3.0	4.4	0.0129	<0.001

Casp4	caspase 4, apoptosis-related cysteine protease	3.0	3.3	0.0011	<0.001
Slc7a8	solute carrier family 7 (cationic amino acid transporter, y+ system), member 8	3.0	4.8	0.0100	<0.001
Tor3a	torsin family 3, member A	3.0	2.2	<0.001	<0.001
Chi3l3	chitinase 3-like 3	3.0	14.8	0.0025	<0.001
	**Down-regulated Genes**				
Temt	thioether S-methyltransferase	-3.4	-6.5	<0.001	<0.001
Myl3	myosin, light polypeptide 3, alkali; ventricular, skeletal, slow	-2.4	-2.0	0.0014	0.0036
