# Supplementary material for: Keratinocytes Determine Th1 Immunity during Early Experimental Leishmaniasis
Source: PLoS Pathog. 2010 Apr 29;6(4):e1000871. doi: 10.1371/journal.ppat.1000871 (PMC2861693; doi:10.1371/journal.ppat.1000871)
Supplement: Table S2 — Genes regulated in C57BL/6 mice (0.03 MB PDF) [file ppat.1000871.s002.pdf]

Table S2. Genes regulated in C57BL/6 mice

MW N-fold = mean regulation compared to uninfected control, TTEST = p-value for regulation in C57BL/6 (C57) or BALB/c (BALB) mice

Gene Symbol	Gene Title	MW N-fold C57	MW N-fold BALB	TTEST C57 reg	TTEST BALB reg
	**Genes Up-regulated**				
Ccr5	chemokine (C-C motif) receptor 5	142.5	8.1	<0.001	0.0024
Cxcl10	chemokine (C-X-C motif) ligand 10	125.7	34.5	0.0022	0.0015
G1p2	interferon, alpha-inducible protein	111.2	27.8	0.0011	0.0014
Fcgr1	Fc receptor, IgG, high affinity I	83.6	30.9	<0.001	0.0012
Irg1	immunoresponsive gene 1	77.8	24.8	<0.001	<0.001
AI447904	expressed sequence AI447904	57.8	8.7	<0.001	0.0013
Slfn4	schlafen 4	52.2	18.4	<0.001	<0.001
Vig1-pending	viral hemorrhagic septicemia virus(VHSV) induced gene 1	47.7	22.5	<0.001	<0.001
Slfn3	schlafen 3	42.8	22.5	<0.001	<0.001
Ms4a6b	membrane-spanning 4-domains, subfamily A, member 6B	42.8	7.7	0.0017	<0.001
Clecsf8	C-type (calcium dependent, carbohydrate recognition domain) lectin, superfamily member 8	40.7	14.1	<0.001	<0.001
Il1b	interleukin 1 beta	39.2	9.8	<0.001	0.0032
Fmnl	formin-like	38.9	10.7	<0.001	0.0055
Usp18	ubiquitin specific protease 18	37.8	16.0	0.0019	<0.001
F10	coagulation factor X	37.0	6.6	0.0021	0.0027
Isg20	interferon-stimulated protein	35.1	31.8	<0.001	0.0016
Ccl4	chemokine (C-C motif) ligand 4	34.6	18.7	<0.001	<0.001
Plac8	placenta-specific 8	30.5	14.0	<0.001	<0.001
Ifit2	interferon-induced protein with tetratricopeptide repeats 2	29.9	25.5	<0.001	<0.001
Ifit1	interferon-induced protein with tetratricopeptide repeats 1	29.7	20.0	<0.001	<0.001
Spp1	secreted phosphoprotein 1	28.3	4.8	<0.001	<0.001
Saa3	serum amyloid A 3	28.1	7.2	<0.001	0.0070
Ccl3	chemokine (C-C motif) ligand 3	28.1	15.0	<0.001	0.0013
Mx1	myxovirus (influenza virus) resistance 1	26.7	30.4	<0.001	<0.001
Ifit3	interferon-induced protein with tetratricopeptide repeats 3	25.4	18.2	<0.001	<0.001
S100a9	S100 calcium binding protein A9 (calgranulin B)	24.6	8.3	<0.001	0.0054
Kdap	kidney-derived aspartic protease-like protein	24.5	14.5	<0.001	<0.001
Slfn2	schlafen 2	24.2	6.4	0.0018	<0.001
Cxcl2	chemokine (C-X-C motif) ligand 2	23.9	12.2	<0.001	<0.001
Adam8	a disintegrin and metalloprotease domain 8	23.1	11.1	<0.001	<0.001
Ifi47	interferon gamma inducible protein	21.9	19.6	<0.001	<0.001
Csf3r	colony stimulating factor 3 receptor (granulocyte)	20.7	16.7	<0.001	<0.001
Itgam	integrin alpha M	20.5	4.8	<0.001	0.0198
Fcer1g	Fc receptor, IgE, high affinity I, gamma polypeptide	20.3	9.7	0.0058	0.0070
S100a8	S100 calcium binding protein A8 (calgranulin A)	18.8	5.7	<0.001	0.0022
Cxcl5	chemokine (C-X-C motif) ligand 5	18.3	9.3	<0.001	<0.001
Tyki	thymidylate kinase family LPS-inducible member	17.4	18.7	<0.001	<0.001
Ccl7	chemokine (C-C motif) ligand 7	16.2	12.0	<0.001	<0.001
Irf7	interferon regulatory factor 7	15.7	10.5	<0.001	<0.001
Basp1	brain abundant, membrane attached signal protein 1	15.3	7.3	<0.001	<0.001
Psmb9	proteosome (prosome, macropain) subunit, beta type 9 (large multifunctional protease 2)	15.2	7.4	<0.001	<0.001
Il6	interleukin 6	15.2	15.7	0.0257	0.0014

Chi3l3	chitinase 3-like 3	14.8	3.0	<0.001	0.0025
Clecsf9	C-type (calcium dependent, carbohydrate recognition domain) lectin, superfamily member 9	14.8	6.5	0.0095	0.0098
Gp49a	glycoprotein 49 A	13.4	8.4	<0.001	<0.001
Ifi204	interferon activated gene 204	13.1	7.7	<0.001	<0.001
Ifi205	interferon activated gene 205	13.1	7.0	<0.001	<0.001
Gbp2	guanylate nucleotide binding protein 2	12.4	9.6	<0.001	<0.001
Mpeg1	macrophage expressed gene 1	12.3	4.2	<0.001	0.0018
Sell	selectin, lymphocyte	12.3	8.4	<0.001	<0.001
Csf2rb1	colony stimulating factor 2 receptor, beta 1, low-affinity (granulocyte-macrophage)	12.2	1.4	<0.001	0.1344
Ccl2	chemokine (C-C motif) ligand 2	12.0	8.7	<0.001	<0.001
H2-T24	histocompatibility 2, T region locus 24	12.0	6.8	<0.001	<0.001
Fpr-rs2	formyl peptide receptor, related sequence 2	12.0	9.2	<0.001	<0.001
Trim30	tripartite motif protein 30	11.9	10.5	<0.001	<0.001
Ccr1	chemokine (C-C motif) receptor 1	11.6	6.1	<0.001	<0.001
Rgl1	ral guanine nucleotide dissociation stimulator,-like 1	11.5	6.5	<0.001	0.0108
Aif1	allograft inflammatory factor 1	11.4	8.2	0.0013	0.0028
Ms4a4c	membrane-spanning 4-domains, subfamily A, member 4C	11.4	7.8	<0.001	<0.001
Ifi203	interferon activated gene 203	11.3	5.3	<0.001	<0.001
Ncf4	neutrophil cytosolic factor 4	11.1	3.6	<0.001	0.0018
Lcn2	lipocalin 2	10.8	5.4	<0.001	<0.001
Cxcr4	chemokine (C-X-C motif) receptor 4	10.6	6.1	0.0012	0.0022
A330075D07	hypothetical protein A330075D07	10.5	6.9	<0.001	<0.001
Slpi	secretory leukocyte protease inhibitor	10.4	2.7	<0.001	<0.001
Cd53	CD53 antigen	10.4	4.7	<0.001	0.0036
Tgtp	T-cell specific GTPase	10.1	18.6	0.0017	<0.001
Tyrobp	TYRO protein tyrosine kinase binding protein	10.0	3.7	<0.001	0.0019
Ccrl2	chemokine (C-C motif) receptor-like 2	9.3	4.6	0.0358	0.0020
Tgfb1	transforming growth factor, beta 1	9.3	2.0	0.0297	0.3524
Itgb2	integrin beta 2	9.1	4.2	<0.001	<0.001
Hdc	histidine decarboxylase	9.0	7.1	<0.001	0.0017
Slfn1	schlafen 1	9.0	6.1	<0.001	<0.001
Hck	hemopoietic cell kinase	8.9	5.2	<0.001	<0.001
Il1rn	interleukin 1 receptor antagonist	8.9	8.8	<0.001	<0.001
Vav1	vav 1 oncogene	8.6	4.1	0.0046	<0.001
Prg	proteoglycan, secretory granule	8.6	5.4	<0.001	<0.001
Cd52	CD52 antigen	8.5	3.8	<0.001	<0.001
Plaur	urokinase plasminogen activator receptor	8.3	2.3	0.0092	0.0074
Nmi	N-myc (and STAT) interactor	8.0	13.0	0.0011	0.0369
Mmp3	matrix metalloproteinase 3	8.0	4.7	<0.001	<0.001
Tnfrsf1b	tumor necrosis factor receptor superfamily, member 1b	7.8	3.7	<0.001	<0.001
Chi3l1	chitinase 3-like 1	7.8	8.8	0.0135	0.0053
Ccl12	chemokine (C-C motif) ligand 12	7.5	5.2	0.0062	<0.001
Timp1	tissue inhibitor of metalloproteinase 1	7.5	4.9	<0.001	<0.001
Hcph	hemopoietic cell phosphatase	7.4	2.0	0.0213	0.0087
Sprr2a	small proline-rich protein 2A	7.3	3.5	<0.001	<0.001
Krt2-5	keratin complex 2, basic, gene 5	7.1	4.8	<0.001	<0.001
9130009C22Rik	RIKEN cDNA 9130009C22 gene	7.0	6.6	<0.001	<0.001
Samhd1	SAM domain and HD domain, 1	6.9	6.0	<0.001	<0.001
Gbp3	guanylate nucleotide binding protein 3	6.4	7.7	0.0010	<0.001
Fes	feline sarcoma oncogene	6.4	2.1	0.0192	<0.001
Ptprc	protein tyrosine phosphatase, receptor type, C	6.2	4.0	0.0042	0.0054

Iigp-pending	interferon-inducible GTPase	6.1	10.1	0.0065	<0.001
Fpr1	formyl peptide receptor 1	6.1	3.2	0.0382	0.1045
Cd83	CD83 antigen	6.0	1.4	0.0269	0.0055
Psmb8	proteosome (prosome, macropain) subunit, beta type 8 (large multifunctional protease 7)	6.0	4.9	<0.001	<0.001
Saa2	serum amyloid A 2	6.0	3.0	<0.001	<0.001
Clecsf6	C-type (calcium dependent, carbohydrate recognition domain) lectin, superfamily member 6	5.9	1.6	0.0204	0.0092
Krt1-16	keratin complex 1, acidic, gene 16	5.9	7.0	0.0030	<0.001
Ifi1	interferon inducible protein 1	5.9	6.2	<0.001	<0.001
Stat1	signal transducer and activator of transcription 1	5.8	5.5	<0.001	<0.001
Lcp1	lymphocyte cytosolic protein 1	5.6	2.0	0.0018	0.0649
Tap1	transporter 1, ATP-binding cassette, sub-family B (MDR/TAP)	5.4	4.6	<0.001	<0.001
Mlp	MARCKS-like protein	5.4	4.5	<0.001	<0.001
Cd14	CD14 antigen	5.3	2.2	<0.001	0.0037
Sprr2b	small proline-rich protein 2B	5.2	1.6	<0.001	0.0020
Bst1	bone marrow stromal cell antigen 1	5.0	2.1	0.0383	0.1593
Sema4d	sema domain, immunoglobulin domain (Ig), transmembrane domain (TM) and short cytoplasmic domain, (semaphorin) 4D	4.9	3.6	<0.001	<0.001
Ninj1	ninjurin 1	4.8	4.3	<0.001	0.0138
Apbb1ip	amyloid beta (A4) precursor protein-binding, family B, member 1 interacting protein	4.8	2.6	<0.001	0.0047
Slc7a8	solute carrier family 7 (cationic amino acid transporter, y+ system), member 8	4.8	3.0	<0.001	0.0100
2010008K16Rik	RIKEN cDNA 2010008K16 gene	4.7	4.2	<0.001	<0.001
Pla2g7	phospholipase A2, group VII (platelet-activating factor acetylhydrolase, plasma)	4.7	3.4	<0.001	0.0080
Slc11a1	solute carrier family 11 (proton-coupled divalent metal ion transporters), member 1	4.6	2.2	<0.001	0.0076
Thbs1	thrombospondin 1	4.6	2.8	<0.001	0.0050
Ctsz	cathepsin Z	4.6	2.7	<0.001	0.0090
Ctss	cathepsin S	4.6	2.5	<0.001	0.0024
Evl	Ena-vasodilator stimulated phosphoprotein	4.6	2.6	<0.001	0.0097
Laptm5	lysosomal-associated protein transmembrane 5	4.6	2.2	0.0021	0.0293
Tlr6	toll-like receptor 6	4.5	2.2	0.0023	0.0017
Igtp	interferon gamma induced GTPase	4.4	6.9	0.0017	<0.001
Gp38	glycoprotein 38	4.4	3.0	<0.001	0.0129
Csf1	colony stimulating factor 1 (macrophage)	4.3	2.9	<0.001	0.0012
Lgals9	lectin, galactose binding, soluble 9	4.3	4.0	<0.001	<0.001
Lcp2	lymphocyte cytosolic protein 2	4.3	3.1	<0.001	<0.001
Pira1	paired-Ig-like receptor A1	4.3	3.3	<0.001	0.0021
Dck	deoxycytidine kinase	4.3	3.5	0.0023	0.0803
Ppicap	peptidylprolyl isomerase C-associated protein	4.3	3.3	<0.001	<0.001
Cyba	cytochrome b-245, alpha polypeptide	4.2	2.1	<0.001	0.0711
F5	coagulation factor V	4.2	2.5	0.0014	0.3247
C3ar1	complement component 3a receptor 1	4.1	2.7	<0.001	0.0047
Msr1	macrophage scavenger receptor 1	4.1	4.7	0.0010	0.0051
Dock2	dedicator of cyto-kinesis 2	4.0	2.2	0.0013	0.0091
Lst1	leukocyte specific transcript 1	4.0	3.9	<0.001	<0.001
Socs3	suppressor of cytokine signaling 3	4.0	3.0	0.0013	<0.001
Csf2rb2	colony stimulating factor 2 receptor, beta 2, low-affinity (granulocyte-macrophage)	4.0	1.8	<0.001	0.0014
Lip1	lysosomal acid lipase 1	4.0	2.4	<0.001	0.0347
Cxcl1	chemokine (C-X-C motif) ligand 1	4.0	3.7	0.0271	0.0031
Apobec1	apolipoprotein B editing complex 1	3.9	3.7	0.0010	<0.001

Lasp1	LIM and SH3 protein 1	3.9	1.8	0.0335	0.0582
Fxyd5	FXYD domain-containing ion transport regulator 5	3.8	1.9	<0.001	0.0199
Ccr2	chemokine (C-C) receptor 2	3.8	2.2	<0.001	0.0104
Lyn	Yamaguchi sarcoma viral (v-yes-1) oncogene homolog	3.8	3.2	<0.001	0.0014
Gtpi-pending	interferon-g induced GTPase	3.8	5.6	<0.001	<0.001
Coro1a	coronin, actin binding protein 1A	3.8	2.2	<0.001	0.0108
Cd68	CD68 antigen	3.8	2.4	<0.001	0.0089
Ms4a4d	membrane-spanning 4-domains, subfamily A, member 4D	3.8	3.8	<0.001	<0.001
Pira3	paired-Ig-like receptor A3	3.7	2.4	<0.001	0.0053
Sp100	nuclear antigen Sp100	3.7	2.8	0.0036	0.0027
Ccl9	chemokine (C-C motif) ligand 9	3.7	1.9	<0.001	0.0075
H2-T17	histocompatibility 2, T region locus 17	3.7	2.5	0.0012	0.0011
Ptafr	platelet-activating factor receptor	3.7	3.7	0.0158	<0.001
Sdcbp	syndecan binding protein	3.6	2.9	<0.001	0.0018
Il7r	interleukin 7 receptor	3.6	2.6	<0.001	<0.001
Tmsb10	thymosin, beta 10	3.6	2.1	<0.001	0.0071
Ncf2	neutrophil cytosolic factor 2	3.6	2.9	<0.001	<0.001
Krt2-6a	keratin complex 2, basic, gene 6a	3.5	3.6	<0.001	0.0028
Unc93b	unc-93 homolog B (C. elegans)	3.5	2.0	0.0011	0.0227
Ogfr	opioid growth factor receptor	3.5	5.4	<0.001	0.0035
Irf1	interferon regulatory factor 1	3.5	3.6	<0.001	<0.001
Scap2	src family associated phosphoprotein 2	3.5	2.3	<0.001	<0.001
Gdap10	ganglioside-induced differentiation-associated-protein 10	3.5	3.3	<0.001	0.0173
Evi2	ecotropic viral integration site 2	3.4	2.8	0.0015	0.0069
Cybb	cytochrome b-245, beta polypeptide	3.4	1.8	0.0067	0.0017
Ftl1	ferritin light chain 1	3.4	2.1	<0.001	0.0128
Lzp-s	P lysozyme structural	3.4	0.7	<0.001	0.2497
Pnp	purine-nucleoside phosphorylase	3.4	3.8	<0.001	<0.001
Isgf3g	interferon dependent positive acting transcription factor 3 gamma	3.4	2.7	<0.001	0.0011
Il10rb	interleukin 10 receptor, beta	3.3	2.0	0.0052	0.0031
Ly86	lymphocyte antigen 86	3.3	1.4	<0.001	0.0446
Myo1f	myosin IF	3.3	2.2	0.0018	0.0027
Casp4	caspase 4, apoptosis-related cysteine protease	3.3	3.0	<0.001	0.0011
Hcls1	hematopoietic cell specific Lyn substrate 1	3.2	2.5	<0.001	0.0045
Tap2	transporter 2, ATP-binding cassette, sub-family B (MDR/TAP)	3.2	3.4	0.0013	<0.001
Ptx3	pentaxin related gene	3.2	5.8	0.0026	0.0098
Gmfg	glia maturation factor, gamma	3.2	2.1	0.0020	0.0085
H2-T10	histocompatibility 2, T region locus 10	3.2	2.3	<0.001	0.0031
Pirb	paired-Ig-like receptor B	3.2	1.8	<0.001	<0.001
1300004C08Rik	RIKEN cDNA 1300004C08 gene	3.1	3.4	<0.001	<0.001
Icam1	intercellular adhesion molecule	3.1	3.8	0.0031	0.0035
Ltb	lymphotoxin B	3.1	3.8	<0.001	0.0016
Wbscr5	Williams-Beuren syndrome chromosome region 5 homolog (human)	3.0	2.0	<0.001	0.0020
Hp	haptoglobin	3.0	2.0	<0.001	<0.001
Psmb10	proteasome (prosome, macropain) subunit, beta type 10	3.0	3.1	0.0017	0.0016
Gsr	glutathione reductase 1	3.0	1.5	0.0158	0.0297
Snx10	sorting nexin 10	3.0	2.2	<0.001	<0.001
Lyzs	lysozyme	3.0	1.3	<0.001	0.2009
Sfpi1	SFFV proviral integration 1	3.0	2.3	<0.001	0.0268

	**Down-regulated Genes**				
Temt	thioether S-methyltransferase	-6.5	-3.4	0.0001	0.0007
Thrsp	thyroid hormone responsive SPOT14 homolog (Rattus)	-4.5	-2.3	0.0558	0.0158
Tbx14	T-box 14	-3.9	-2.0	0.0015	0.0100
Cdo1	cysteine dioxygenase 1, cytosolic	-3.4	-2.8	0.0015	0.0002
Ereg	epiregulin	-3.2	-1.0	0.0017	0.0703
Sprr2h	small proline-rich protein 2H	-2.9	-1.3	0.0006	0.0941
Nov	nephroblastoma overexpressed gene	-2.8	-1.9	0.0003	0.0005
1110031K21Rik	RIKEN cDNA 1110031K21 gene	-2.7	-1.8	0.0317	0.0269
Myoc	myocilin	-2.6	-1.8	0.0008	0.0021
Enpp2	ectonucleotide pyrophosphatase/phosphodiesterase 2	-2.5	-1.6	<0.0001	0.0036
Tbx15	T-box 15	-2.5	-2.1	0.0063	0.0035
Amot	angiomotin	-2.4	-1.9	<0.0001	0.0063
Fgfbp1	fibroblast growth factor binding protein 1	-2.3	-1.8	<0.0001	0.0020
Sparcl1	SPARC-like 1 (mast9, hevin)	-2.3	-1.6	0.0005	0.0248
Ank1	ankyrin 1, erythroid	-2.2	-2.0	0.0002	0.0025
Myl2	myosin, light polypeptide 2, regulatory, cardiac, slow	-2.2	-1.9	<0.0001	0.0011
