# Supplementary material for: Keratinocytes Determine Th1 Immunity during Early Experimental Leishmaniasis
Source: PLoS Pathog. 2010 Apr 29;6(4):e1000871. doi: 10.1371/journal.ppat.1000871 (PMC2861693; doi:10.1371/journal.ppat.1000871)
Supplement: Table S3 — Genes which were stronger regulated in BALB/c mice (0.01 MB PDF) [file ppat.1000871.s003.pdf]

Table S3. Genes which were stronger regulated in BALB/c mice

MW N-fold = mean regulation compared to uninfected control, TTEST = p-value for differential regulation in BALB/c versus C57BL/6 (C57) mice.

Gene Symbol	Gene Title	MW N-fold Balb	MW N-fold C57	TTEST
	**Genes Up-regulated**			
Gbp1	guanylate nucleotide binding protein 1	18.0	-1.6	0.0007
Ifi202b	interferon activated gene 202B	5.2	1.8	0.0010
Klra2	killer cell lectin-like receptor, subfamily A, member 2	16.5	4.5	0.0274
Ptx3	pentaxin related gene	5.8	3.2	0.0280
