# Supplementary material for: Keratinocytes Determine Th1 Immunity during Early Experimental Leishmaniasis
Source: PLoS Pathog. 2010 Apr 29;6(4):e1000871. doi: 10.1371/journal.ppat.1000871 (PMC2861693; doi:10.1371/journal.ppat.1000871)
Supplement: Table S4 — Genes which were stronger regulated in C57BL/6 mice (0.02 MB PDF) [file ppat.1000871.s004.pdf]

Table S4. Genes which were stronger regulated in C57BL/6 mice
MW N-fold = mean regulation compared to uninfected control, TTEST = p-value for differential regulation in C57BL/6 (C57) versus BALB/c (BALB) mice.

Gene Symbol	Gene Title	MW N-fold C57	MW N-fold BALB	TTEST
	**Genes Up-regulated**			
Ccr5	chemokine (C-C motif) receptor 5	142.5	8.1	0.0058
Csf2rb1	colony stimulating factor 2 receptor, beta 1, low-affinity (granulocyte-macrophage)	12.2	1.4	0.0143
AI447904	expressed sequence AI447904	57.8	8.7	0.0123
Spp1	secreted phosphoprotein 1	28.3	4.8	0.0012
F10	coagulation factor X	37.0	6.6	0.0251
Ms4a6b	membrane-spanning 4-domains, subfamily A, member 6B	42.8	7.7	0.0446
Lzp-s	P lysozyme structural	3.4	0.7	0.0135
Chi3l3	chitinase 3-like 3	14.8	3.0	<0.001
Tgfb1	transforming growth factor, beta 1	9.3	2.0	0.0487
Cd83	CD83 antigen	6.0	1.4	0.0416
Itgam	integrin alpha M	20.5	4.8	0.0202
Il1b	interleukin 1 beta	39.2	9.8	0.0337
Saa3	serum amyloid A 3	28.1	7.2	0.0373
Slpi	secretory leukocyte protease inhibitor	10.4	2.7	0.0027
Clecsf6	C-type (calcium dependent, carbohydrate recognition domain) lectin, superfamily member 6	5.9	1.6	0.0179
S100a8	S100 calcium binding protein A8 (calgranulin A)	18.8	5.7	<0.001
Sprr2b	small proline-rich protein 2B	5.2	1.6	<0.001
Irg1	immunoresponsive gene 1	77.8	24.8	0.0184
S100a9	S100 calcium binding protein A9 (calgranulin B)	24.6	8.3	0.0035
Mpeg1	macrophage expressed gene 1	12.3	4.2	0.0031
Clecsf8	C-type (calcium dependent, carbohydrate recognition domain) lectin, superfamily member 8	40.7	14.1	0.0059
Slfn4	schlafen 4	52.2	18.4	0.0093
Lcp1	lymphocyte cytosolic protein 1	5.6	2.0	0.0231
Sprr2a	small proline-rich protein 2A	6.3	2.3	0.0015
Tyrobp	TYRO protein tyrosine kinase binding protein	10.0	3.7	0.0168
Cd14	CD14 antigen	5.3	2.2	0.0029
Lyzs	lysozyme	3.0	1.3	0.0028
Ly86	lymphocyte antigen 86	3.3	1.4	0.0045
Cd52	CD52 antigen	8.5	3.8	0.0092
Cd53	CD53 antigen	10.4	4.7	0.0149
Plac8	placenta-specific 8	30.5	14.0	0.0223
Csf2rb2	colony stimulating factor 2 receptor, beta 2, low-affinity (granulocyte-macrophage)	4.0	1.8	0.0076
Itgb2	integrin beta 2	9.1	4.2	0.0013
Tnfrsf1b	tumor necrosis factor receptor superfamily, member 1b	7.8	3.7	<0.001
Fcgr2b	Fc receptor, IgG, low affinity IIb	2.7	1.3	0.0013
Basp1	brain abundant, membrane attached signal protein 1	15.3	7.3	0.0116
Slc11a1	solute carrier family 11 (proton-coupled divalent metal ion transporters), member 1	4.6	2.2	0.0013
Adam8	a disintegrin and metalloprotease domain 8	23.1	11.1	0.0072
Cyba	cytochrome b-245, alpha polypeptide	4.2	2.1	0.0125
Gsr	glutathione reductase 1	3.0	1.5	0.0071
Fxyd5	FXYD domain-containing ion transport regulator 5	3.8	1.9	0.0060
Lcn2	lipocalin 2	10.8	5.4	0.0376
Cxcl2	chemokine (C-X-C motif) ligand 2	23.9	12.2	0.0035
Ptpns1	protein tyrosine phosphatase, non-receptor type	2.8	1.5	0.0065

	substrate 1			
Ccl9	chemokine (C-C motif) ligand 9	3.7	1.9	0.0076
Ccr1	chemokine (C-C motif) receptor 1	11.6	6.1	0.0179
Slfn3	schlafen 3	42.8	22.5	0.0377
Ifi205	interferon activated gene 205	13.1	7.0	0.0015
Cybb	cytochrome b-245, beta polypeptide	3.4	1.8	<0.001
Ctss	cathepsin S	4.6	2.5	0.0349
Apbb1ip	amyloid beta (A4) precursor protein-binding, family B, member 1 interacting protein	4.8	2.6	0.0255
Dock2	dedicator of cyto-kinesis 2	4.0	2.2	0.0232
BC032204	cDNA sequence BC032204	2.5	1.4	0.0029
Ccr2	chemokine (C-C) receptor 2	3.8	2.2	0.0046
1110007C09Rik	RIKEN cDNA 1110007C09 gene	2.7	1.5	0.0128
Evl	Ena-vasodilator stimulated phosphoprotein	4.6	2.6	0.0110
	**Genes Down-regulated**			
Sprr2h	small proline-rich protein 2H	-2.9	-1.3	0.0049
Temt	thioether S-methyltransferase	-6.5	-3.4	0.0022
Tbx14	T-box 14	-3.9	-2.0	0.0421
