# Supplementary material for: Keratinocytes Determine Th1 Immunity during Early Experimental Leishmaniasis
Source: PLoS Pathog. 2010 Apr 29;6(4):e1000871. doi: 10.1371/journal.ppat.1000871 (PMC2861693; doi:10.1371/journal.ppat.1000871)
Supplement: Table S5 — Absolute expression levels of genes analyzed by Real-time PCR in uninfected skin of BALB/c and C57BL/6 mice (expressed as copy number/10000 copies of glyceraldehyde-3-phosphate dehydrogenase (GAPDH)) (0.01 MB PDF) [file ppat.1000871.s005.pdf]

Table S5. Absolute expression levels of genes analyzed by *Real-time* PCR in uninfected skin of BALB/c and C57BL/6 mice (expressed as copy number/ 10000 copies of glyceraldehyde-3-phosphate dehydrogenase (GAPDH))

Gene	Mean copy number/ 10000 copies GAPDH BALB/c	SE copy number	Mean copy number/ 10000 copies GAPDH C57BL/6	SE copy number
**IL-1ß**	1,900	0,541	15,871	4,721
**IL-4**	0,020	0,005	0,020	0,003
**IL-6**	0,596	0,002	0,371	0,031
**IL-10**	1,667	0,982	1,972	0,002
**IL-12p35**	0,522	0,247	0,878	0,212
**IL-12p40**	0,738	0,302	0,980	0,614
**IL-13**	2,679	0,872	1,365	0,076
**TGF-b**	28,905	11,113	28,106	19,654
**TNF-a**	2,734	1,332	3,700	0,691
**Opn**	31,328	9,068	29,617	18,235
**CXCL1**	0,695	0,048	2,546	1,996
**CXCL2**	0,771	0,005	1,055	0,068
**CXCL9**	1,300	1,029	0,916	0,692
**CXCL10**	6,604	4,614	3,348	3,165
**CCL2**	4,594	3,628	3,757	2,134
**CCL3**	0,441	0,258	0,197	0,089
**CCL4**	0,920	0,458	0,409	0,110
**CCL5**	7,471	4,193	6,898	2,234
**CCL7**	5,843	2,360	3,434	2,774
**CCR1**	3,854	0,958	5,920	1,447
**CCR2**	7,005	1,085	12,807	1,628
**CCR5**	2,006	0,087	4,068	1,197
**Temt**	0,954	0,024	0,883	0,0351
**Sprr2h**	2,887	1,065	6,068	0,335
**SLPI**	5,997	1,065	8,036	0,334
**MRP8**	14,213	8,147	12,547	4,999
**MRP14**	6,345	0,521	22,370	4,734
**F4/80**	17,212	2,580	26,521	15,312
**Ym-1**	0,887	0,001	1,544	0,098
**Sprr2a**	2,054	0,887	2,001	1,065
