# Supplementary material for: Keratinocytes Determine Th1 Immunity during Early Experimental Leishmaniasis
Source: PLoS Pathog. 2010 Apr 29;6(4):e1000871. doi: 10.1371/journal.ppat.1000871 (PMC2861693; doi:10.1371/journal.ppat.1000871)
Supplement: Table S6 — Functional clusters overrepresented in genes regulated in C57BL/6 mice (0.01 MB PDF) [file ppat.1000871.s006.pdf]

Table S6. Functional clusters overrepresented in genes regulated in C57BL/6 mice.

Functional gene clusters overrepresented among genes up-regulated in C57BL/6 mice	Z Score	P-value	% of selection	% of all
**molecular function**				
chemokine activity	12.064	<0.001	5.47	0.37
IgG binding	8.641	<0.001	1.49	0.06
cytokine activity	8.004	<0.001	9.45	1.89
receptor activity	4.574	<0.001	19.90	10.25
signal transducer activity	6.95	0	36.8	18.2
hematopoietin/interferon-class (D200-domain) cytokine receptor activity	3.657	0.007	2.49	0.57
interleukin receptor activity	3.377	0.022	1.49	0.27
hematopoietin/interferon-class (D200-domain) cytokine receptor binding	2.926	0.02	1.49	0.33
hydrolase activity	2.015	0.045	19.9	14.9
**biological process**				
immune response	20.297	<0.001	43.28	7.08
chemotaxis	15.939	<0.001	13.43	1.23
humoral immune response	9.233	<0.001	9.45	1.54
neutrophil chemotaxis	8.931	<0.001	2.49	0.14
phagocytosis	8.879	<0.001	3.48	0.27
antigen presentation	6.4	<0.001	3.98	0.59
JAK-STAT cascade	6.338	<0.001	2.99	0.36
cytokine production	5.72	<0.001	4.48	0.84
myeloid cell differentiation	5.276	<0.001	3.98	0.77
cytokine and chemokine mediated signaling pathway	5.138	<0.001	2.49	0.36
keratinization	5.107	0.001	1.99	0.24
cell-mediated immune response	4.054	0.002	2.49	0.50
apoptosis	3.833	0.002	11.44	5.41
positive regulation of cytokine production	3.352	0.014	1.99	0.44
T-helper 1 type immune response	3.19	0.01	1.99	0.47
T cell activation	2.83	0.02	2.99	1.01
I-kappaB kinase/NF-kappaB cascade	2.419	0.03	2.49	0.90
