# Supplementary material for: Keratinocytes Determine Th1 Immunity during Early Experimental Leishmaniasis
Source: PLoS Pathog. 2010 Apr 29;6(4):e1000871. doi: 10.1371/journal.ppat.1000871 (PMC2861693; doi:10.1371/journal.ppat.1000871)
Supplement: Table S7 — Functional clusters overrepresented in genes regulated in Balb/c mice (0.01 MB PDF) [file ppat.1000871.s007.pdf]

Table S7. Functional clusters overrepresented in genes regulated in BALB/c mice.

Functional gene clusters overrepresented among genes up-regulated in BALB/c mice	Z Score	P-value	% of selection	% of all
**molecular function**				
chemokine activity	14.328	<0.001	7.48	0.37
cytokine activity	10.508	<0.001	13.61	1.90
signal transducer activity	5.864	<0.001	36.73	18.25
hematopoietin/interferon-class (D200-domain) cytokine receptor binding	4.987	<0.001	2.72	0.34
receptor activity	2.698	0.009	17.01	10.31
hydrolase activity	2.378	0.017	21.77	14.87
**biological process**				
immune response	17.366	<0.001	43.54	7.11
chemotaxis	13.77	<0.001	13.61	1.23
JAK-STAT cascade	6.254	<0.001	3.40	0.36
nitric oxide biosynthesis	6.171	0.002	2.72	0.24
neutrophil chemotaxis	6.159	<0.0010	2.04	0.14
cytokine production	5.254	<0.0010	4.76	0.84
humoral immune response	5.193	<0.0010	6.80	1.56
cellular defense response	5.103	<0.0010	5.44	1.10
myeloid cell differentiation	4.637	0.001	4.08	0.77
keratinization	4.477	0.004	2.04	0.24
phagocytosis	4.168	0.005	2.04	0.27
apoptosis	3.7	<0.0010	12.24	5.41
cytokine and chemokine mediated signaling pathway	3.459	0.015	2.04	0.36
antigen presentation	3.43	0.011	2.72	0.59
T cell activation	2.92	0.022	3.40	1.01
