# Supplementary material for: Keratinocytes Determine Th1 Immunity during Early Experimental Leishmaniasis
Source: PLoS Pathog. 2010 Apr 29;6(4):e1000871. doi: 10.1371/journal.ppat.1000871 (PMC2861693; doi:10.1371/journal.ppat.1000871)
Supplement: Table S8 — Absolute expression levels of genes analyzed by Real-time PCR in keratinocytes isolated from uninfected skin of BALB/c and C57BL/6 mice (expressed as copy number/10000 copies of glyceraldehyde-3-phosphate dehydrogenase (GAPDH)) (0.01 MB PDF) [file ppat.1000871.s008.pdf]

Table S8. Absolute expression levels of genes analyzed by *Real-time* PCR in keratinocytes isolated from uninfected skin of BALB/c and C57BL/6 mice (expressed as copy number/ 10000 copies of glyceraldehyde-3-phosphate dehydrogenase (GAPDH))

Gene	Mean copy number/ 10000 copies GAPDH BALB/c	SEM copy number	Mean copy number/ 10000 copies GAPDH C57BL/6	SEM copy number
**IL-1ß**	7,646	3,558	0,670	0,327
**IL-4**	0,038	0,017	0,040	0,013
**IL-6**	1,078	0,889	0,903	0,471
**IL-10**	4,687	2,730	3,721	2,100
**IL-12p40**	1,883	1,033	0,632	0,291
**TGF-b**	0,316	0,142	1,801	1,278
**TNF-a**	8,724	5,054	3,173	1,682
**Opn**	47,673	17,290	67,588	32,388
**CXCL2**	0,056	0,006	0,008	0,004
**CXCL9**	0,309	0,033	1,464	0,875
**CXCL10**	0,309	0,033	0,502	0,268
**CCL2**	6,745	2,756	0,557	0,259
**CCL3**	2,353	1,315	1,509	0,751
**CCL4**	0,346	0,236	0,194	0,059
**CCL5**	3,911	2,129	0,947	0,445
**CCL7**	0,020	0,008	2,002	0,040
**CCR1**	1,966	0,006	1,050	0,040
**CCR2**	0,044	0,002	13,540	4,891
**Temt**	3,054	2,518	0,464	1,995
**MRP 8**	4,888	2,978	3,449	2,006
**MRP 14**	86,208	51,173	97,412	52,600
**Sprr2a**	0,584	0,004	2,443	0,289
