# Supplementary material for: Keratinocytes Determine Th1 Immunity during Early Experimental Leishmaniasis
Source: PLoS Pathog. 2010 Apr 29;6(4):e1000871. doi: 10.1371/journal.ppat.1000871 (PMC2861693; doi:10.1371/journal.ppat.1000871)
Supplement: Table S9 — cDNA sequences used as templates for RISH (0.01 MB PDF) [file ppat.1000871.s009.pdf]

Table S9. cDNA sequences used as templates for RISH

Gene	Genebank Accession	Nucleotides
CXCL2	NM_009140.1	13-1024
CXCL10	M86829.1	9-849
Ym-1	NM_009892.1	1-1385
TNFα	gi|7305584	15-1537
opn	NM_009263	102-987
