# Supplementary material for: Keratinocytes Determine Th1 Immunity during Early Experimental Leishmaniasis
Source: PLoS Pathog. 2010 Apr 29;6(4):e1000871. doi: 10.1371/journal.ppat.1000871 (PMC2861693; doi:10.1371/journal.ppat.1000871)
Supplement: Table S10 — Primers used for Real-time PCR (0.06 MB PDF) [file ppat.1000871.s010.pdf]

Table S10. Primers used for Real-time PCR

Oligonucleotide	Sequence
CCL2F (MCP-1)<br>CCL2R	5’-TTG ACC CGT AAA TCT GAA GCT AAT-3’<br>5’-TCA CAG TCC GAG TCA CAC TAG TTC AC-3’
CCL3F (MIP-1α)<br>CCL3R	5’-CGA GTA CCA GTC CCT TTT CTG TTC-3’<br>5’-AAG ACT TGG TTG CAG AGT GTC ATG-3’
CCL4F (MIP-1β)<br>CCL4R	5’-AAG CTG CCG GGA GGT GTA AG-3’<br>5’-TGT CTG CCC TCT CTC TCC TCT TG-3’
CCL5F (RANTES)<br>CCL5R	5’-TGC CCA CGT CAA GGA GTA TTT C-3’<br>5’-TCC TAG CTC ATC TCC AAA TAG TTG ATG-3’
CCL7F (MCP-3)<br>CCL7R	5’-GGA TCT CTG CCA CGC TTC TG-3’<br>5’-TCC TTC TGT AGC TCT TGA GAT TCC TC-3’
CCR1F<br>CCR1R	5’-AGT ACC TTC GGC AGC TGT TTC A-3’<br>5’-TTT CTA GTT GGT CCA CAG AGA GGA A-3’
CCR2F<br>CCR2R	5’-CATGCAAGTTCAGCTGCCTG-3’<br>5’-TGCCGTGGATGAACTGAGG-3’
CCR5F<br>CCR5R	5’-GTCTATGGATGTGCCTGACTGC-3’<br>5’-CCGATTTACCCCAAGTTCTGC-3’
CXCL1F (GRO-α)<br>CXCL1R	5’-GCG AAA AGA AGT GCA GAG AGA TAG AG-3’<br>5’-CGT GCG TGT TGA CCA TAC AAT ATG-3’
CXCL2F (MIP-2)<br>CXCL2R	5’-GTC CCT CAA CGG AAG AAC CAA-3’<br>5’-ACT CTC AGA CAG CGA GGC ACA T-3’
CXCL9F (MIG)<br>CXCL9R	5’-ATT GTG TCT CAG AGA TGG TGC TAA TG-3’<br>5’-TGA AAT CCC ATG GTC TCG AAA G-3’
CXCL10F (IP-10)<br>CXCL10R	5’-CCT AGC TCA GGC TCG TCA GTT CT-3’<br>5’-GGA AGA TGG TGG TTA AGT TTG TCC TT-3’
GAPDH3F<br>GAPDH3R	5’-GGT CCT CAG TGT AGC CCA AGA TG-3’<br>5’-TGG CCT TCC GTG TTC CTA CC-3’
F4/80F<br>F4/80R	5’-CTG TAA CCG GAT GGC AAA CTT G-3’<br>5’-ACA CAG CAG GAA GGT GGC TAT G-3’
IL-1βF<br>IL-1βR	5’-TGT CTT GGC CGA GGA CTA AGG-3’<br>5’-TGG GCT GGA CTG TTT CTA ATG C-3’
IL-4F<br>IL-4R	5’-CAC GGA TGC GAC AAA AAT CAC-3’<br>5’-GAG GAC GTT TGG CAC ATC CA-3’
IL-6F<br>IL-6R	5’-TGA GAT CTA CTC GGC AAA CCT AGT G-3’<br>5’-CTT CGT AGA GAA CAA CAT AAG TCA GAT ACC-3’
IL-10F<br>IL-10R	5’-GGG TTG CCA AGC CTT ATC G-3’<br>5’-TCT CAC CCA GGG AAT TCA AAT G-3’

IL-12p40F<br>IL-12p40R	5’-CCT AAG TTC ATC ATG ACA CCT TTG C-3’<br>5’-CCA AGT GGA ATG CTA GAA TAT CTA TGC-3’
IL-13 F<br>IL-13R	5’-GCC TGT TAC ACT CAA GGT GAT GTG-3’<br>5’-CTG TCA CCA TCT TTA TTT CCG GTT T-3’
Rpl13aF (ribosomal protein L)<br>Rpl13aR	5’-TGG TCC CTG CTG CTC TCA AG-3<br>5’-GGC CTT TTC CTT CCG TTT CTC-3’
S100A8F (S100 calcium binding proteinA8)<br>S100A8R	5’-ATC ACC ATG CCC TCT ACA AGA ATG-3’<br>5’-GTC CAA TTC TCT GAA CAA GTT TTC G-3’
S100A9F (S100 calcium binding protein A9)<br>S100A9R	5`-GAG GGC TTC ATT TCT CTT CTC TTT C-3’<br>5’-TCA TCG ACA CCT TCC ATC AAT ACT C-3’
SLPIF (secretory leukocyte protease inhibit.)<br>SLPIR	5’-GTG CTG TGA GGG TAT ATG TGG-3’<br>5’-GCG CCA ATG TCA GGG ATC AG-3’
OPNF (Osteopontin, secreted phosphoprotein 1)<br>OPNR	5’-AGC TTT ACA GCC TGC ACC CAG-3’<br>5’-TTC TTC AGA GGA CAC AGC ATT CTG-3’
Sprr2aF (small proline rich protein 2a)<br>Sprr2aR	5’-GGTGAAGGAGAACGACCAGAAG-3’<br>5’-TCAACTGTACCCAGGTGCCC-3’
Sprr2hF (small proline rich protein 2h)<br>Sprr2hR	5’-TGG ATA CTC TTC TCT TCC CCT TGT C-3’<br>5’-TCC CTC ATA CCA GCA GAA ATG C-3’
TemtF (thioether S-methyl-transferase)<br>TemtR	5’-GAC CTC GCA TGC TGA AAG CA-3’<br>5’-CAG AGC AGG AAA TCG TAA AGT TTA GC-3’
TGF-βF (transforming growth factor β)<br>TGF-βR	5’-GGA CCC TGC CCC TAT ATT TGG-3’<br>5’-TGT TGC AGG TCA TTT AAC CAA GTG-3’
TNF-αF (tumor necrosis factor α)<br>TNF-αR	5’-AGA AAC ACA AGA TGC TGG GAC AGT-3’<br>5’-CCT TTG CAG AAC TCA GGA ATG G-3’
Ym-1F (Chi3l3, chitinase 3-like 3, Ym-1)<br>Ym-1R	5’-GGA GTA GAG ACC ATG GCA CTG AAC-3’<br>5’-GAC TTG CGT GAC TAT GAA GCA TTG-3’
